# Ultralow contact resistance in organic transistors via orbital hybridization

**DOI:** 10.1038/s41467-023-36006-0

**Published:** 2023-01-19

**Authors:** Junpeng Zeng, Daowei He, Jingsi Qiao, Yating Li, Li Sun, Weisheng Li, Jiacheng Xie, Si Gao, Lijia Pan, Peng Wang, Yong Xu, Yun Li, Hao Qiu, Yi Shi, Jian-Bin Xu, Wei Ji, Xinran Wang

**Affiliations:** 1 National Laboratory of Solid State Microstructures, School of Electronic Science and Engineering, and Collaborative Innovation Center of Advanced Microstructures, Nanjing University, Nanjing, 210093 P. R. China; 2 MIIT Key Laboratory for Low-Dimensional Quantum Structure and Devices, School of Integrated Circuits and Electronics, Beijing Institute of Technology, Beijing, 100081 China; 3 Department of Physics and Beijing Key Laboratory of Optoelectronic Functional Materials & Micro-nano Devices, Renmin University of China, Beijing, 100872 P. R. China; 4 National Laboratory of Solid State Microstructures, Jiangsu Key Laboratory of Artificial Functional Materials, College of Engineering and Applied Sciences and Collaborative Innovation Center of Advanced Microstructures, Nanjing University, Nanjing, P. R. China; 5 College of Electronic and Optical Engineering, Nanjing University of Posts and Telecommunications, Nanjing, P. R. China; 6 Department of Electronic Engineering and Materials Science and Technology Research Center, The Chinese University of Hong Kong, Hong Kong SAR, P. R. China; 7 School of Integrated Circuits, Nanjing University, Suzhou, P. R. China

**Keywords:** Molecular electronics, Electronic devices

## Abstract

Organic field-effect transistors (OFETs) are of interest in unconventional form of electronics. However, high-performance OFETs are currently contact-limited, which represent a major challenge toward operation in the gigahertz regime. Here, we realize ultralow total contact resistance (*R*_c_) down to 14.0 Ω ∙ cm in C_10_-DNTT OFETs by using transferred platinum (Pt) as contact. We observe evidence of Pt-catalyzed dehydrogenation of side alkyl chains which effectively reduces the metal-semiconductor van der Waals gap and promotes orbital hybridization. We report the ultrahigh performance OFETs, including hole mobility of 18 cm^2^ V^−1^ s^−1^, saturation current of 28.8 μA/μm, subthreshold swing of 60 mV/dec, and intrinsic cutoff frequency of 0.36 GHz. We further develop resist-free transfer and patterning strategies to fabricate large-area OFET arrays, showing 100% yield and excellent variability in the transistor metrics. As alkyl chains widely exist in conjugated molecules and polymers, our strategy can potentially enhance the performance of a broad range of organic optoelectronic devices.

## Abstract (web summary)

The limitation in metal-semiconductor contact has been a major challenge for high-performance organic field-effect transistors. Here, the authors fabricate the contact by transferring platinum electrode on solution-processed organic films, realizing ultralow total contact resistance down to 14 Ω ∙ cm.

## Introduction

Organic electronics featuring flexibility and lightweight is a promising technology for a range of applications such as foldable displays, plastic tags, and artificial skin^1–5^. Over the past 30 years, tremendous progress has been made to improve organic device performance through designing high-mobility molecules and understanding the charge transport mechanism^6–9^. Despite these advances, however, one of the remaining fundamental issues in organic devices has been the metal-semiconductor (M-S) contact^3,10,11^. Currently, the lowest *R*_c_ in OFETs is several tens of Ω⋅cm^3,11,12^, which is at least two orders of magnitude higher than competing inorganic technologies based on, for example, oxide semiconductors and two-dimensional (2D) materials^13–15^. The large *R*_c_ can be attributed to the following reasons. (i) At the atomic level, the metal-molecule contact is van der Waals (vdW) in nature, which is distinct from covalently bonded contact in inorganic semiconductors^16,17^. The vdW gap causes the decoupling of electronic states between metals and molecules and greatly reduces the carrier injection efficiency, similar to the case of 2D semiconductors^13^. (ii) Conventional deposition process involves high-energy metal ions bombarding the organic film which leads to a high density of chemical and structural disorders^1,18^. The metal-induced gap states result in Fermi-level pinning and a large Schottky barrier regardless of materials combinations. To further improve *R*_c_ beyond the state-of-the-art, both issues need to be addressed simultaneously. In particular, the electronic coupling between the molecular and metallic orbitals needs to be enhanced by rational theoretical design and experimental implementation that preserves the integrity of the contact interface.

Here, we realized ultralow *R*_c_ in OFETs by enhancing M-S orbital hybridization in several classes of high-performance organic semiconductors (including C_10_-DNTT, C_8_-BTBT, and Ph-BTBT-C_10_). The contact was fabricated by transfer of Pt electrode on solution-processed monolayer organic films to ensure direct contact with the conducting layer and pristine interface. We observed evidence of dehydrogenation of side alkyl chains catalyzed by Pt, thereby reducing the vdW gap and enhancing the metal-molecule orbital hybridization, as corroborated by first-principles calculations. Compared with the non-catalytic Au contact, Pt exhibited more than four times lower *R*_c_ (as low as 14 Ω·cm) and 20% enhancement in current density. As a result, the intrinsic cutoff frequency of our OFETs is estimated to 0.36 GHz, which took a major step toward gigahertz organic transistors^10^. Furthermore, the large-area OFET arrays exhibited excellent variability and long-term air stability, which made our approach scalable for circuit-level applications.

## Results

### Ultralow contact resistance using transferred Pt contact

**Fig. 1:**
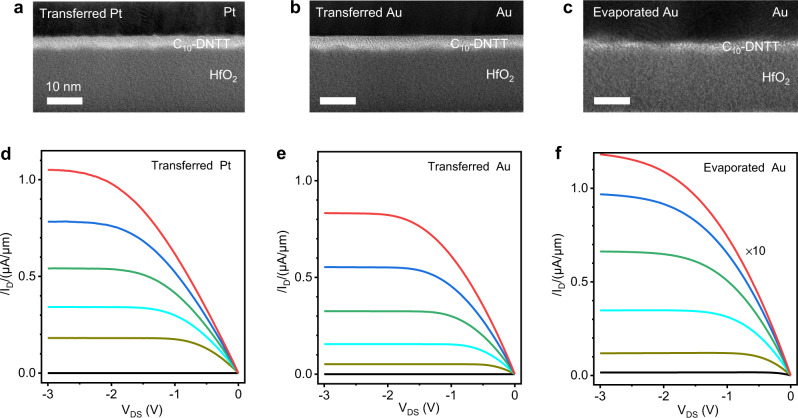
Characterizations of metal and organic semiconductor contact. Cross-sectional TEM image of metal/monolayer C_10_-DNTT/HfO_2_ stacks by transferring Pt (**a**) and Au (**b**) electrode, and evaporating Au (**c**) electrode on top of C_10_-DNTT, respectively. Typical room temperature *I*_D_~*V*_DS_ characteristics of transferred-Pt (**d**), transferred-Au (**e**) and evaporated-Au (**f**) devices with the channel length/width of 30/112, 30/122, and 30/800 μm), respectively. From top to bottom in **d** and **e** (**f**), *V*_GS_ = −3.0 V (−3.0 V), −2.65 V (−2.5 V), −2.3 V (−2.0 V), −1.95 V (−1.5 V), −1.6 V (−1.0 V) and −0.55 V (−0.5 V), respectively.

We used a solution shearing process to grow centimeter-size monolayer C_10_-DNTT films in ambient condition^19^ (see Methods for details). The domain size could reach several millimeters, which removed the influence of domain boundary and improved the device uniformity. To form high-quality contact, we mechanically peeled the pre-patterned Pt electrodes on Si substrate using PMMA and gently laminated on onto the organic films (see Methods). Pt has a work function of 5.6 eV, which matches the ionization potential (IP) of C_10_-DNTT (5.4 eV) and is expected to form p-type Ohmic contact^20^. We first performed scanning transmission electron microscopy (STEM) characterization of the contact interface. Figure 1a, b shows the typical cross-section STEM images of contact interface with laminated Pt and Au, respectively, both showing atomically smooth and sharp interface without metal-induced disorders^21^. The pristine interface was maintained across the entire contact region (Supplementary Fig. 1). The thickness of C_10_-DNTT in was 3.9 nm, corresponding to monolayer (Supplementary Fig. 2a). On the other hand, the thermally evaporated-Au introduced notable damage to the monolayer organic semiconductor due to the high-energy ion bombardment (Fig. 1c). Figure 1d–f plot the corresponding output (I_D_~V_DS_) characteristics of the three types of OFETs. Among them, the transferred-Pt OFETs exhibited the highest current density (measured drain current divided by channel width (*W*)), indicating the best electrical contact. In contrast, the evaporated-Au device showed nearly 10 times lower current due to the large contact resistance.

**Fig. 2:**
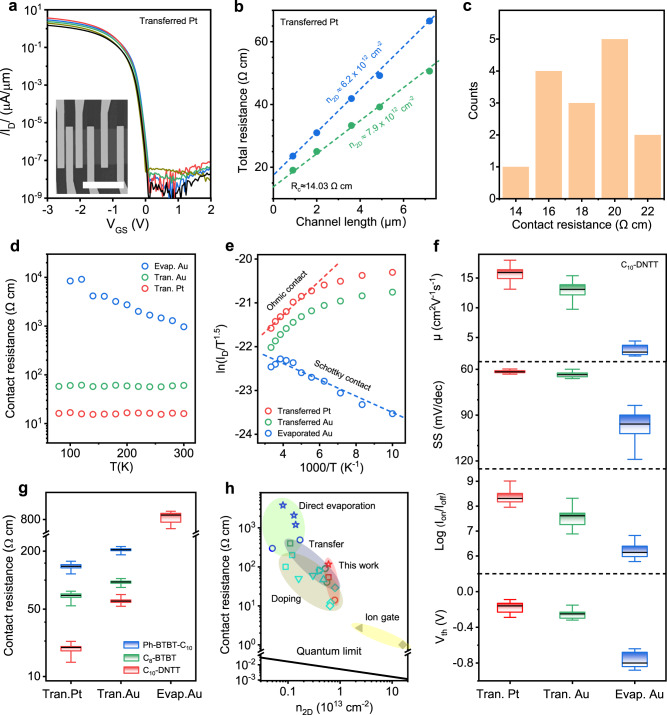
Electrical contact properties of transferred-Pt, transferred-Au, and evaporated-Au monolayer C_10_-DNTT OTFTs. **a** Room temperature I_D_~V_GS_ characteristics of a typical TLM structure of transferred-Pt OTFTs with a channel length of 0.9, 2.0, 3.6, 4.6, and 7.2 μm under V_DS_ = −1.0 V. The TLM structure has the same channel width of 27 μm. Inset shows the scanning electron microscope (SEM) image of TLM structure. Scale bar, 30 μm. **b** *R*_c_ extraction using TLM method from the device in **a**. **c** Histogram of the contact resistance of transferred-Pt OTFTs. **d** The *R*_c_ as a function of temperature in three types of OFETs. The contact resistances in **c**, **d** were extracted at V_DS_ = −1.0 V and *n*_2*D*_ = 7.9 × 10^12^ cm^−2^. **e** Arrhenius plots of the Ohmic transferred-Pt (red open circle) and transferred-Au (green open circle) OTFTs at V_GS_ = −3.0 V and V_DS_ = −0.3 V, and Schottky evaporated OFETs (blue open circle) at *V*_GS_ = −3.0 V and V_DS_ = −1.0 V, respectively. The channel length and width see Supplementary Figs. 5 and 7. **f** Statistics of effective mobility (*μ*), subthreshold swing (SS), on/off ratio (*I*_on_/*I*_off_), and threshold voltage (*V*_th_) of 42 transferred-Pt, 16 transferred-Au, and 21 evaporated-Au contact C_10_-DNTT OFETs with channel length more than 10 μm at room temperature. The average values are 15.8 (13.0/2.9) cm^2^ V^−1^ s^−1^, 61.6 (63.6/96.6) mV/decade, 2.24 × 10^8^ (3.6 × 10^7^/1.8 × 10^6^), −0.18 (−0.26/−0.75) V, respectively, for transferred-Pt (transferred-Au/evaporated-Au) contact. The corresponding best values see Supplementary Table 2. **g** *R*_c_ distribution of several kinds of ultrathin organic crystalline films with three contact types of OTFTs with channel lengths ranging from 0.9 to 45 μm, respectively. **f**, **g** The black lines represent the average of statistical data. **h** State-of-the-art contact technology for OFETs plotted as a function of carrier concentration, showing the optimal *R*_c_ of various organic semiconductors. The solid black line represents the quantum limit for contact resistance. For references, see Supplementary Table 1. The symbols in **h** represent C_10_-DNTT (open circle), C_8_-BTBT (open square), Ph-BTBT-C_10_ (open star), C_8_-DNBDT-NW (open up triangle), C_9_-DNBDT-NW (open down triangle), Dph-DNTT (open diamond), IDTBT (open pentagon), Pentacene (open triangle), P3HT (solid diamond) and PDPP (solid left triangle), respectively.

To reveal the advantage of Pt contact, we extracted *R*_c_ by transfer-length-method (TLM) structure with channel length (*L*) ranging from 0.6 to 20 μm (see Methods for more details). The *I*_D_~*V*_GS_ transfer characteristics were measured in the linear regime, and the R_tot_~*L* linear-regression lines was plotted under the same carrier concentration (n_2D_), where the y-intercept, x-intercept, and slope correspond to *R*_c_, 2*L*_T_ (*L*_T_ is the transfer length) and sheet resistance (*R*_*sheet*_), respectively^13,22^. Figure 2a shows typical room temperature *I*_D_~*V*_GS_ characteristics of a TLM structure with transferred-Pt contact. From TLM, we deduced *R*_c_ = 14.0 Ω∙cm and *L*_T_ = 0.9 μm at *n*_2*D*_ = 7.9 × 10^12^ cm^−2^ and *V*_DS_ = −1.0 V (Fig. 2b, Supplementary Fig. 3), which were, to the best of our knowledge, one of the lowest records in OFETs (Supplementary Table 1). Statistical analysis of 15 TLM devices showed average *R*_c_ of 19.4 ± 2.2 Ω∙cm (Fig. 2c), which was 3.1 times and 44 times lower than the transferred-Au and evaporated-Au contact, respectively (Supplementary Table 1). The correlation coefficient *R*^2^ (>0.99) and standard deviation *σ* (error bars) from the fitting process indicated that the derived *R*_c_ was reliable and reproducible (Supplementary Fig. 4).

Next, we performed variable-temperature measurements to evaluate the contact properties. The transferred-Pt exhibited several signatures of Ohmic contact. (i) The *R*_c_ was nearly independent of temperature within our experimental range (Fig. 2d). (ii) The *I*_D_~*V*_DS_ characteristics maintained good linearity down to 100 K, and the current was enhanced at low temperature, showing intrinsic band-like carrier transport (Supplementary Figs. 5 and 6). (iii) Arrhenius plot (${{{{\mathrm{ln}}}}}(\tfrac{{I}_{D}}{{T}^{1.5}}) \sim \tfrac{1000}{T}$) showed positive slopes (Fig. 2e), which corresponded to negative Schottky barrier height (*ϕ*_*B*_)^13,16^. In contrast, the evaporated-Au contract showed non-Ohmic behavior (Fig. 2d, e), and *ϕ*_*B*_ = 18.8 meV was extracted from the flat-band model (Supplementary Fig. 7). Devices with evaporated-Au contact also showed degraded subthreshold swing, effective mobility, on/off ratio and positive threshold voltage shift (Fig. 2f), indicating that these devices were contact-limited.

We also study the generality of the transferred Pt to reduce *R*_c_ in OFETs. To this end, we fabricated TLM structures based on C_8_-BTBT and Ph-BTBT-C_10_, which were high-mobility oligomers with alkyl side chains. Supplementary Figs. 8–10 show the representative electrical characteristics. In both cases, we observed Ohmic contact behavior and intrinsic band-like transport properties similar to C_10_-DNTT. The average *R*_c_ was 67.0 and 139.2 Ω∙cm for C_8_-BTBT and Ph-BTBT-C_10_, respectively, which was ~2 times lower than transferred-Au contact (Fig. 2g). Figure 2h summarizes the *R*_c_ of OFETs using different contact technologies. It shows that the transferred-Pt contact delivers record-low *R*_c_ for all the same materials used in this study, suggesting that it can effectively enhance the performance of a broad range of organic devices.

### Mechanistic insight of low contact resistance

**Fig. 3:**
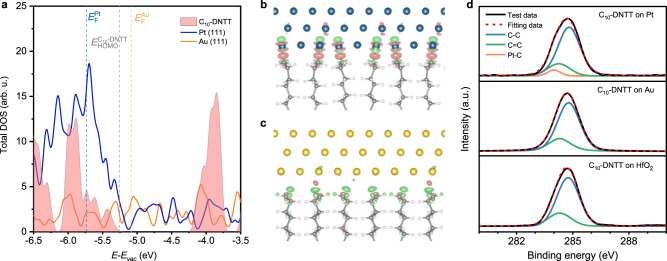
Mechanism of C_10_-DNTT and metal contact. **a** Total DOS for 1L C_10_-DNTT (pink filled region), Pt (111) (blue solid line), and Au (111) (yellow solid line). E_F_^Pt^_,_ E_F_^Au^_,_ E_HOMO_^C10-DNTT^ mark the Fermi energy and the energy of valence band maximum for Pt (111), Au (111), and 1 L C_10_-DNTT, respectively. **b**, **c** Differential charge density between C_10_-DNTT and Pt (**b**) and Au (**c**) electrodes using an isosurface of 0.005 and 0.001 e bohr^−3^, respectively. Pink and green colors represent charge accumulation and reduction, respectively. **d** XPS spectra of C_10_-DNTT on HfO_2_, Au, and Pt film substrate, respectively. a.u. is arbitrary units.

The observed superior contact properties of transferred Pt require further theoretical understanding. One possibility is that the higher work function of Pt leads to lower *ϕ*_*B*_. However, this can be ruled out as both transferred Pt and Au showed negative *ϕ*_*B*_ from low-temperature measurements (Fig. 2e). To shed light on the mechanism, we performed density functional theory (DFT) calculations on the electronic properties of C_10_-DNTT/Pt and C_10_-DNTT/Au contacts (see Methods for calculation details). In C_10_-DNTT, the two highest occupied molecular orbitals responsible for the hole transport (HOMO and HOMO-1) are the anti-bonding and bonding states of S-C-S, which are contributed by the DNTT core rather than associated alkyl chains (Supplementary Fig. 11). When forming electrical contact, the alkyl chains between DNTT and the metal layer act as a tunneling barrier. Figure 3a shows the total density of states (DOS) of C_10_-DNTT, Pt (111), and Au (111). Pt (111) shows much higher DOS than Au (111) within the energy range of −5.3 ~ −6.0 eV, corresponding to the energy level of HOMO and HOMO-1 states of C_10_-DNTT. This indicates that the hole-type carriers of Pt (111) have a larger probability of tunneling from Pt than from Au to C_10_-DNTT, leading to lower contact resistance.

Furthermore, in the C_10_-DNTT/Pt, we observed dehydrogenated alkyl chains forming covalent bonds with Pt (111) in the equilibrium state with −3.17 eV Pt-C bond energy and 2.09 Å bond length, in the typical covalent bond energy range. The dehydrogenation of other alkane molecules was also observed above certain temperatures on Pt (111), e.g. methane at 120 K^23,24^. The carbon‐hydrogen bond strength in a methane (CH_4_) molecule is the strongest among methyl group. Thus, the dehydrogenated alkyl chains forming covalent bonds with Pt happened when Pt was transferred on C_10_-DNTT at room temperature. The covalently bonded interface could further enhance the tunneling probability between C_10_-DNTT and Pt (Fig. 3b and Supplementary Fig. 12). In contrast, dehydrogenation was not observed in C_10_-DNTT/Au contact. As shown in Fig. 3c, the C_10_-DNTT/Au interface was primarily vdW in nature, and the binding energy (9 meV/Å^2^) was smaller than the vdW binding energy of graphene/Au (28 meV/Å^2^)^25^ (Fig. 3c and Supplementary Fig. 12).

We experimentally verified the DFT calculations using X-ray photoemission spectroscopy (XPS). Figure 3d plots the XPS spectra and fitting of C_10_-DNTT deposited on Pt, Au and HfO_2_, respectively. The C 1*s* peaks of C_10_-DNTT on HfO_2_ and Au could be deconvoluted into two peaks at 284.8 eV and 284.4 eV, which are labeled as C-C (sp^3^) and C = C (sp^2^), respectively (see Methods for more details). For C_10_-DNTT on Pt, the XPS peak width was wider due to an additional side peak at 284.1 eV, corresponding to Pt-C bonds^26–28^. Evidence of Pt-C covalent bond was also observed in C_8_-BTBT and Ph-BTBT-C_10_ (Supplementary Fig. 13). These results provided strong support of Pt-catalyzed dehydrogenation of side alkyl chains, which effectively reduced the M-S vdW gap and promotes orbital hybridization as shown in Fig. 3b.

To further confirm the advantage of Pt-catalyzed dehydrogenation at the contact interface, we compared the current density of C_10_-DNTT and pentacene OFETs with transferred-Pt and -Au contact, where the pentacene dehydrogenation on Pt surface is unable to happen at room temperature^29,30^. Therefore, the observed enhancement of current density in transferred-Pt pentacene OFETs is mainly from the higher work function of Pt (Supplementary Fig. 14), just one-third of C_10_-DNTT devices. For C_10_-DNTT/Pt contact, the charge carriers were injected into the conjugated core of C_10_-DNTT by tunneling through the vdW gap and the remaining portion of the alkyl chain. The shorter alkyl chain could provide the higher charge injection efficiency^31^, significantly improving the contact resistance, as shown in Supplementary Fig. 15 that compares contact resistance of C_6_-DNTT, C_10_-DNTT, C_14_-DNTT transferred-Pt OFETs. However, these two tunneling processes have different carrier injection efficiency due to the different dielectric constant and tunneling barrier^32–35^. Therefore, we counterintuitively observed the contact resistance 14.0 Ω∙cm at *n*_2*D*_ = 7.9 × 10^12^ cm^−2^ with the total tunneling gap of 11 Angstroms^32^. In short, the improvement of *R*_c_ and current in C_10_-DNTT/Pt contact stems from better orbital hybridization induced by Pt-catalyzed dehydrogenation and perfect energy match.

### High-performance and high-frequency OFETs

**Fig. 4:**
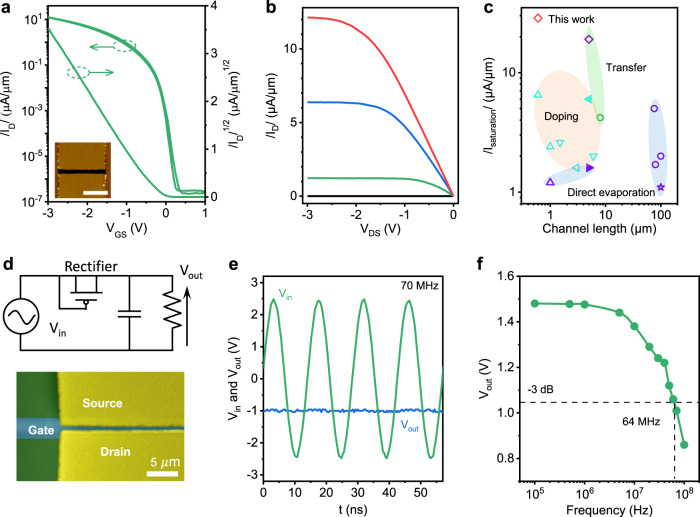
Ultrahigh performance of transferred-Pt OTFTs. **a** *I*_D_~*V*_GS_ characteristics of typical transferred-Pt monolayer C_10_-DNTT OFET with channel length/width of 0.65/10 μm under *V*_DS_ = −3.0 V. Inset shows the AFM image of the device. Scale bar: 5 μm. **b** *I*_D_~*V*_DS_ characteristics of the device in **a**. From top to bottom, *V*_GS_ = −3.0 V, −2.0 V, −1.0 V, and 0 V, respectively. **c** The on-state saturation current as a function of channel length for various OFETs made by the state-of-the-art contact technology. The symbols in **c** represent C_6_-DNTT (open circle), Ph-BTBT-C_10_ (open star), C_8_-DNBDT-NW (open left triangle), C_9_-DNBDT-NW (open down triangle), Dph-DNTT (open up triangle), Tips-pentacene (solid right triangle) and C_12_-BTBT (solid left triangle), respectively. References in **c** see Supplementary Table 3. **d** The a.c.- d.c. rectifying circuit (up) and optical microscope (down) of a typical diode-connected OTFT with channel length/width of 0.7/180 μm, gate-to-source overlap of 1.1 μm, and gate-to-drain overlap of 1.2 μm. **e** The input a.c. carrier signal and output d.c. voltage as the frequency of 70 MHz. The amplitude of the input sinusoidal-wave voltage of 2.5 V and the load capacitor of 82 nF. **f** The output d.c. voltage as a function of frequency.

To fully unveil the potential of transferred-Pt contact, we fabricated scaled C_10_-DNTT OFETs with sub-1 μm channel length. Figure 4a, b plot the *I*_D_~*V*_GS_ and *I*_D_~*V*_DS_ characteristics of a representative device with *L* = 600 nm. The device exhibited several remarkable properties, including linear *I*_D_~*V*_GS_ characteristics at low bias, large on-state current of 12.1 μA/μm, subthreshold swing of 60 mV/dec, near-zero threshold voltage, on/off ratio close to 10^8^ and negligible hysteresis. To remove the self-heating effect, we performed pulse I-V measurements in C_10_-DNTT and C_6_-DNTT OFETs, which the largest on-state current was improved to 15.8 and 28.6 μA/μm (Supplementary Fig. 16 and Table 3). We benchmark the on-state current with existing high-performance OFETs using doped, transferred, and evaporated contacts, as summarized in Fig. 4c and Supplementary Table 3. Our device exhibited the highest on-state current to date, showing 34% improvement over the previous record under similar channel length^3,12,19,31,36^.

The combination of ultralow *R*_c_ and short channel allowed us to realize high-performance radio-frequency devices. We first estimated the intrinsic cutoff frequency, ${f}_{T,intrinsic}=\tfrac{{\upsilon }_{sat}}{2\pi L}$, where *υ*_*sat*_, *L* are saturation velocity and channel length, respectively^16^. *υ*_*sat*_ was extracted from the *I*_D_~*V*_DS_ characteristics by $\upsilon=\tfrac{1}{W\,{C}_{i}}\tfrac{\partial {I}_{D}}{\partial {V}_{GS}}$ according to the gradual channel approximation^16,37^ (see Methods). For *L* = 0.6 μm devices, the *υ*_*sat*_ = 1.35 × 10^5 ^cm/s and *f*_*T*, *intrinsic*_ = 0.36 GHz was estimated. We note that the *f*_*T*,*intrinsic*_ could be potentially boosted above GHz by further scaling *L*. We next fabricated a diode-connected OFET (also called coplanar rectifier), which was an important component in high-frequency applications. Compared to vertical organic diode rectifier, coplanar rectifier is more appealing because it could reduce parasitic capacitance induced by electrode overlap and restrain excess Joule heating^11,38–40^.

Figure 4d shows a diode-connected OFET rectifier with channel length of 700 nm fabricated on highly resistive silicon substrates. The dynamic performance was accessed by applying a sinusoidal input with an amplitude of 2.5 V under different frequency (Fig. 4e and Supplementary Fig. 17). After rectifying, we gained a d.c. output voltage of 1.48 and 1.0 V at a frequency of 1 and 70 MHz, respectively. We defined the maximum rectifying frequency (*f*_*R*_) at −3 dB output voltage^41,42^, which can be expressed by ${f}_{R}=\tfrac{\mu {V}_{a.c.}}{2{L}^{2}}(\tfrac{\sqrt{1-{\beta }^{2}}}{{\cos }^{-1}\beta }-\beta )$, where *β* is $\tfrac{{V}_{out}}{{V}_{a.c.}}$, *V*_*a.c*._ is the amplitude of input a.c. signal. The experimentally measured *f*_*R*_ was 64 MHz at V_a.c._ = ±2.5 V (Fig. 4f), which was about a halve of calculated *f*_*R*_ = 0.13 GHz that was estimated at *β* = 0.42, *μ* = 1.32 cm^2^ V^−1^ s^−1^, *V*_*a.c*._ = 2.5 *V* and *L* = 0.7 μm, respectively. The main cause of such discrepancy may be the gate parasitic capacitance induced by the overlap of gate and source/drain electrodes^35^. The normalized experimental frequency of 25.6 MHz/V was the highest yet reported among diode-connected OFET rectifiers (Supplementary Table 4).

### Large-area device array and variability

**Fig. 5:**
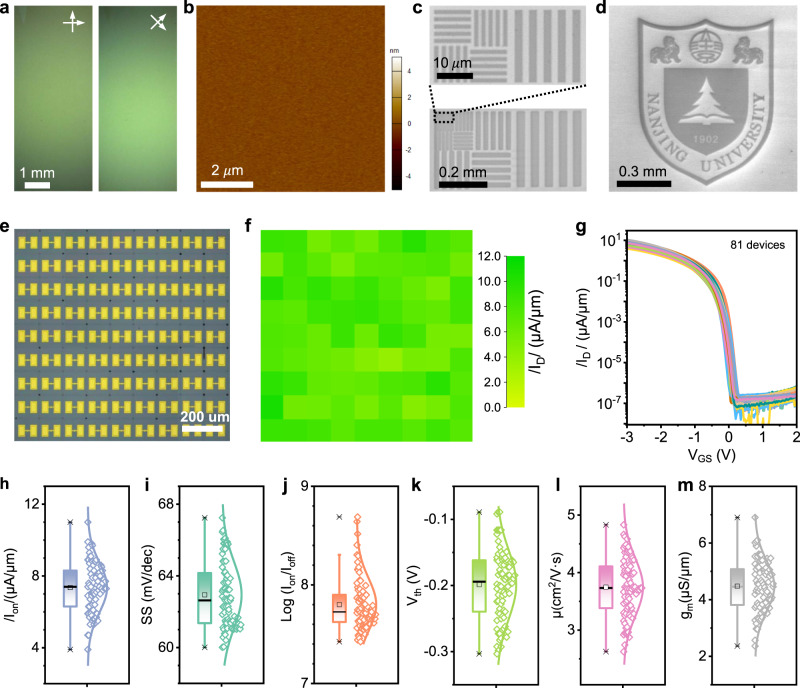
C_10_-DNTT OTFT array performance. **a** Cross-polarized optical microscopy of uniform monolayer C_10_-DNTT single crystal, showing a single crystal except for a small portion at the top. **b** Atomic force microscopy image of monolayer C_10_-DNTT films. **c**, **d** Scanning electron micrograph of electron-beam generated line and NJU logo pattern of monolayer C_10_-DNTT film, respectively. The blackened area is irradiated by the electron beam. **e** Photograph of a fabricated 9 × 9 OFET array with a channel length/width of 0.6/10 μm. **f** Distribution of saturation current density at *V*_DS_ = −3 V and *V*_GS_ = −3 V. **g** *I*_D_~*V*_GS_ characteristics of 81 OFETs in **e**. **h**–**m** Statistical distribution of saturation current density, subthreshold swing, on-off ratio, threshold voltage, effective mobility and channel transconductance of the device in **a**, respectively.

To assess the scalability of our approach, we grew centimeter scale crystalline C_10_-DNTT films and fabricated OFET arrays. The single crystallinity was confirmed by cross-polarized optical microscopy (Fig. 5a, b). The absence of domain boundary could further improve the device performance and uniformity. For large-area arrays or integrated circuits, microscale patterning of organic thin-film is critical prerequisite. To this end, we directly subjected the organic film to electron-beam irradiation in an electron-beam lithography tool, where the exposed areas became pyrolyzing and insulating (Supplementary Fig. 18) while the non-exposed areas remained unchanged and did not have the fringe effect (Supplementary Fig. 19)^43,44^. We were able to write lines and complex patterns with sub-micrometer resolution (Fig. 5c, d).

Next, we fabricated OFETs array with density of 1.1 × 10^4^ OFETs per square centimeter. We used spin-coated large-area PMMA to transfer metal arrays onto the pre-pattered organic films, with micrometer resolution (see Methods and Supplementary Fig. 20 for more details). Figure 5e–g shows the microscope image, current distribution, and transfer characteristics of 9 × 9 array with channel length of 0.6 μm. Owing to the high-quality of C_10_-DNTT film and scalable metal transfer technique, we achieved 100% yield and 15% on-state current variation (the ratio of the standard deviation over mean value), where the highest and average on-state current was 11.1 μA/μm and 7.4 μA/μm, respectively (Fig. 5h). Figure 5i–m show statistical analysis of subthreshold swing, on/off ratio, threshold voltage, effective mobility and transconductance of the 81 OFETs. The best values were 60.1 mV/dec, 8.7 × 10^8^, −0.089 V, 4.7 cm^2^ V^−1^ s^−1^, and 6.9 μS/μm, and the variations were 5.4%, 1.3%, 0.9%, 13%, 15%, respectively. Furthermore, we performed the stability measurement on transferred-Pt-C_10_-DNTT OFETs. The result showed excellent air stability over 3 months with almost no degradation (Supplementary Fig. 21).

## Discussion

In conclusion, our transferred-Pt OFETs exhibited several hallmark features, including ultralow *R*_c_ of 14.0 Ω∙cm, hole mobility of 18 cm^2^ V^−1^ s^−1^, saturation current of 28.8 μA/μm and subthreshold swing of 60 mV/dec. On the benefit of these excellent electrical properties, the intrinsic cutoff frequency of our transferred-Pt OFET was estimated to 0.36 GHz. This strategy is scalable, allowing us to fabricate large-area arrays with high yield and reproducibility. As alkyl chains widely exist in conjugated molecules and polymers, our strategy can potentially enhance the performance of a broad range of organic optoelectronic devices. We anticipate our work to further advance the organic semiconductors towards GHz devices.

## Methods

### ALD of HfO_2_ dielectrics

Atomic layer deposition (ALD) was performed to deposit 20 nm HfO_2_ film on p-doped silicon substrate at 150 °C and a base pressure of ~1 Pa using tetrakis (dimethylamido) hafnium and H_2_O as precursors. 20 sccm N_2_ was used as carrier gas. The pulse/purge times for Hf and H_2_O precursors were 300 ms/30 s and 30 ms/30 s, respectively. The capacitance of 20 nm HfO_2_ is about 5.1 × 10^−7^ Fcm^−2^.

### Growth and characterization of ultrathin crystalline thin-film

The thickness and morphology were carefully characterized by atomic force microscopy (Asylum Cypher). We further finely optimized the growth temperature, blade shear speed, and surface roughness of substrate, leading to the creation of centimeter-level scale single crystal monolayer film that could be enough to fabricate several hundreds of OFETs. In this paper, the thickness of all referred C_10_-DNTT crystalline thin-film is about 4 nm, corresponding to monolayer C_10_-DNTT. Using this approach, we also got the large-area single crystalline C_8_-BTBT and Ph-BTBT-C_10_ thin-film.

We used cross-polarized optical microscopy to characterize the crystalline of monolayer C_10_-DNTT film by the ScanPro spectro-microscope under the white light at ambient condition.

The cross-sectional TEM specimens were prepared using the standard FEI Scois Dualbeam focused ion beam. After that, the specimens were characterized using a high-resolution JEOL JEM-2100F TEM with acceleration voltage of 200 kV equipped with energy- dispersive spectroscopy detector.

We used electron-beam irradiation to generate the pattern of monolayer C_10_-DNTT film by the electron-beam lithography (EBL) under the dose of about 700 C · cm^2^ and 30 KeV electron-beam. After that, we snap these patterns by scanning transmission electron microscopy at 10 KeV electron-beam.

The XPS characterization was performed using a Thermo Fisher Scientific Escalab 250Xi instrument with Al K*α* X-ray source at a power of 150 W and a spot size of 500 μm. The spectra were acquired with operation voltage of 12.5 kV and the spectrometer pressure of 8 × 10^−10^ mbar. We obtained all of the spectra under the identical conditions. All of the XPS spectra were calibrated by the peak of C 1s at 284.8 eV. And the spectrum decomposition was performed using the XPS peak 41 programs with Lorentzian-Gaussian functions after subtraction of a Shirley-Linear background.

### Monolayer OTFT fabrication and integration

The pre-patterned metal electrodes, including 120 nm Au and 100/20 nm Au/Pt, were deposited on a silicon substrate using electron-beam lithography (EBL) and electron-beam evaporation (EBE).

For single OTFT, two patches were picked up and transferred onto the monolayer C_10_-DNTT film sequentially as source/ drain electrodes. The more details of the transfer process were described by He et al.^45^.

For OTFT array, a PMMA layer was spin-coated onto the large-area pre-patterned metal arrays, followed by the lamination of thermal released tape (TRT). The TRT/PMMA/metal stack was carefully released from silicon substrate and laminated onto the surface of large-area monolayer C_10_-DNTT film. Then the TRT was released at 100 °C with a very slowly temperature rising process and subsequently the OTFT array was patterned to single device by EBL that could completely separate the adjacent two OTFTs. For device test, we open a window on the source and drain electrode by standard EBL.

For diode-connected OFET rectifier, we first deposited 20 nm HfO_2_ film on high resistivity silicon substrates, and then deposited 15 nm Ti/Au as gate electrode, subsequently deposited another 20 nm HfO_2_ film as gate dielectrics. Meanwhile, we opened a window on to the gate electrode for connecting the source or drain electrode. Next, the C_10_-DNTT films were grown and the source/drain electrodes were laminated onto their surface. At last, we connected the gate and source/drain electrodes by metal lead.

### Electrical measurements

All Electrical measurements were carried out by an Agilent B1500 semiconductor parameter analyzer in a closed-cycle probe station with a base pressure of 10^−5^ torr. The stability measurement device was stored at ambient condition and tested in closed-cycle condition.

Further, the rectifier characteristics of diode-connected OFET rectifier was measured that a function generator (Tektronix AFG31000 SERIES) was used to apply an input carrier signal and an oscilloscope (Tektronix MDO3034) was applied to read the input and output voltages.

### Contact resistance and intrinsic mobility

The reduction of *R*_c_ (including source resistance and drain resistance in main text) not only decreases the power supply voltage but also increases the operation frequency of OFETs in application of organic circuits. *R*_c_ in OFETs includes access resistance (*R*_*access resistance*_) that comes from the vertical tunneling barrier (dependence on the thickness of organic film between contact metal and current conduction layer) and interface resistance (*R*_*interface resistance*_) that is exponential proportional to interfacial Schottky barrier ($\exp (\tfrac{{\varPhi }_{B}}{{k}_{B}T})$^1^. Therefore, the total resistance (R_total_) includes *R*_c_ and channel resistance (*R*_*ch*_), expressed by *R*_*total*_ = *R*_*c*_ + *R*_*ch*_ = (*R*_*access resistance*_ + *R*_*interface resistance*_*)* + *R*_*sheet*_ * *L*, where *R*_*sheet*_ (The unit is KΩ per square) and *L* are sheet resistance and channel length of organic semiconductor channel, respectively. In our devices, the *R*_*total*_ and *R*_*sheet*_ are spatially homogeneous in linear regime. Therefore, the total resistance has a linear dependance with *L*. As shown in Fig. 2a, the total *R*_*total*_ is extracted at *L* = 0 and *R*_*shee*t_ is the slope at a certain carrier concentration. The intrinsic mobility of our devices can be calculated by ${\mu }_{intrinsic}=\tfrac{1}{q{n}_{2D}{R}_{sheet}}$.

### Quantum limit of electrical contact resistance

Assuming ballistic transport in ideal M-S junction, there exists a fundamental quantum limit in junction resistance, expressed by: ${R}_{C,min}W=\tfrac{h}{2{q}^{2}}{e}^{2{k}_{0}d}\sqrt{\tfrac{\pi }{2{g}_{v}{n}_{s}}}$, where *h* is Plank’s constant, q is the unit charge, *g*_v_ is valley degeneracy, *n*_s_ the carrier concentration, *d* is the size of vdW gap (Do not include the alkyl chain of the organic semiconductor) and *k*_0_ is the decay constant, respectively. The *k*_0_ is equal to $\sqrt{\tfrac{2{m}^{\ast }{\varphi }_{avg}}{{\hslash }^{2}}}$, where *φ*_*avg*_ is tunneling energy barrier, which relays on work function of contact metal and electron affinity or ionization energy^35^.

### Extraction of Schottky barrier

When a metal is in close proximity to organic semiconductor surface, both Fermi levels line up and Schottky barrier (Φ_*SB*_), energy difference between the work function of the metallic electrode and the electron affinity or ionization potential of the semiconductor, is formed at the interface. Therefore, we could think of the ultrathin C_10_-DNTT OFETs as two back-to-back connected Schottky diodes that the thermally emission of low-bias current form the contact electrode into single-layer molecule channel can be expressed by thermionic emission, ${I}_{D}\propto {T}^{1.5}\exp (-\tfrac{q{\varPhi }_{B}}{{k}_{B}T})$, where *T* is temperature, *q* is the electronic charge, *k*_*B*_ is the Boltzmann constant, and Φ_*B*_ is the Schottky barrier height^13,14,16^. From the linear fitting Arrhenius plot in Supplementary Fig. 7, we extracted barrier height under different gate voltage, which is separated into two regimes by the flat-band voltage. Below the flat-band voltage, the barrier height has a linear dependence on gate voltage due to thermionic emission dominating. At the flat-band voltage, thermally assisted tunneling become relevant, the barrier height starts to deviate from the linear trend, and tunneling will dominate the current injection. The Schottky barrier height, 18.8 meV, is extracted at the flat-band voltage.

### Saturation velocity

In the main text, all three types of contacts show excellent linearity in output characteristics at low bias, and nearly complete current saturation about *V*_DS_ = 1.5 V, which is one of the lowest saturation voltages among OFETs^46–48^. The phenomenon of saturated current is more likely from carrier velocity saturation due to the transverse gate electric field far more than the longitudinal source–drain electric field in our devices^16^. By gradual channel approximation, the on-state current can be modeled by ${{{{{{\rm{I}}}}}}}_{{{{{{\rm{D}}}}}}}=\tfrac{{{{{{\rm{W}}}}}}}{{{{{{\rm{L}}}}}}}{\int }_{0}^{{{{{{\rm{L}}}}}}}|{{{{{\rm{Q}}}}}}({{{{{\rm{y}}}}}})|{{{{{\rm{v}}}}}}({{{{{\rm{y}}}}}}){{{{{\rm{dy}}}}}}={{{{{{\rm{vWC}}}}}}}_{{{{{{\rm{i}}}}}}}({{{{{{\rm{V}}}}}}}_{{{{{{\rm{GS}}}}}}}-{{{{{{\rm{V}}}}}}}_{{{{{{\rm{th}}}}}}})$, where *Q*(y), *W*, *L*, *C*_i_ are carrier density, channel width, channel length and gate capacitance, respectively. By differentiating with respect to the gate voltage, the expression of carrier velocity is obtained: ${{{{{{\rm{v}}}}}}}_{{{{{{\rm{drift}}}}}}}=\tfrac{1}{{{{{{{\rm{WC}}}}}}}_{{{{{{\rm{i}}}}}}}}\tfrac{\partial {{{{{{\rm{I}}}}}}}_{{{{{{\rm{D}}}}}}}}{\partial {{{{{{\rm{V}}}}}}}_{{{{{{\rm{GS}}}}}}}}$.

### DFT calculations

DFT calculations were performed using the generalized gradient approximation for the exchange-correlation potential, the projector augmented wave method^49,50^, and a plane-wave basis set as implemented in the Vienna ab-initio simulation package^51^. In all calculations, vdW interactions were considered in the framework of vdW-DF with the optB86b functional for the exchange energy (optB86b-vdW)^52,53^, which was found suitable for modeling geometric and electronic properties in 2D organic materials^54–56^. The energy cutoff for the plane-wave basis was set to 600 eV in the simulation of C_10_-DNTT, and was set to 400 eV for C_10_-DNTT/Pt or Au contact systems. A k-mesh of 8 × 6 × 1 was used to sample the Brillouin zone of C_10_-DNTT layers. The vacuum region is ~20 Å in thickness. The shape and volume of C_10_-DNTT layers were fully relaxed until the residual force per atom was less than 0.01 eV/Å. In terms of contact systems, a 1 × 2 C_10_-DNTT supercell with a vacuum layer of >15 Å was used and the lattice constants were kept as that of C_10_-DNTT. A k-mesh of 6 × 4 × 1 was used to sample the Brillouin zone. The shape and volume of the supercell and below four-layers metal atoms were kept fixed. All other atoms were fully relaxed until the residual force per atom was less than 0.02 eV/Å. The energy convergence criteria for all self-consistent cycles was 1 × 10^−5 ^eV.

## Supplementary information

Supplementary Information

## Data Availability

Source data are provided in this paper.

## Source data

Source Data
