# Supplementary material for: Ultralow contact resistance in organic transistors via orbital hybridization
Source: Nat Commun. 2023 Jan 19;14:324. doi: 10.1038/s41467-023-36006-0 (PMC9852566; doi:10.1038/s41467-023-36006-0)
Supplement: Supplementary file 1 — Supplementary Information [file 41467_2023_36006_MOESM1_ESM.pdf]

Supplementary Information

Ultralow contact resistance in organic transistors via orbital hybridization

Junpeng Zeng,[1,†] Daowei He,[1,*,†] Jingsi Qiao,[2,3,*,†] Yating Li,[1,†] Li Sun,[1] Weisheng Li,[1] Jiacheng Xie,[1] Si Gao,[4] Lijia Pan,[1] Peng Wang,[4] Yong Xu,[5] Yun Li,[1] Hao Qiu,[1] Yi Shi,[1] Jian-Bin Xu,[6] Wei Ji,[3] and Xinran Wang[1,7,8,*]

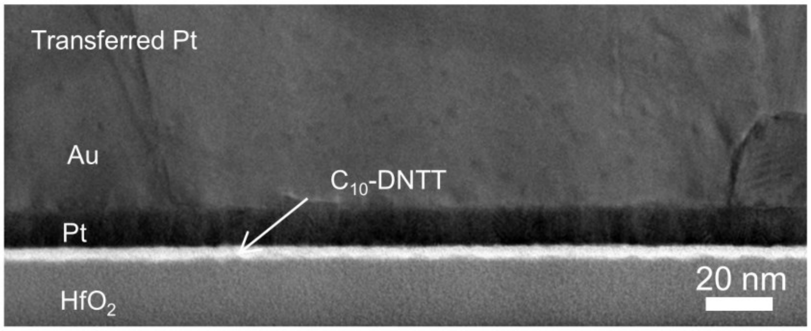

Supplementary Figure 1. Large-area STEM image of metallic electrodes/C_{10}-DNTT/HfO_2 stack.

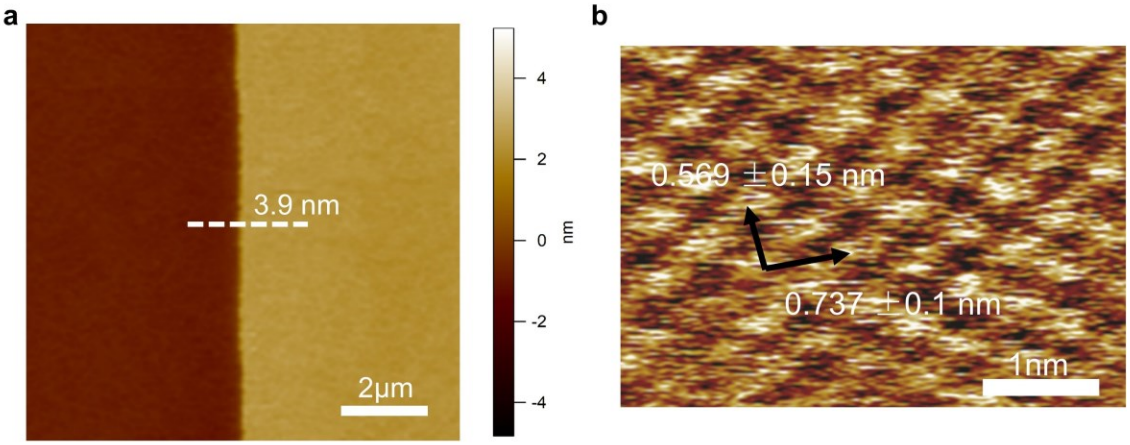

Supplementary Figure 2. AFM images of monolayer C_{10}-DNTT (**a**) and high-resolution

AFM image (**b**). The monolayer crystalline films adopt molecular herringbone-type packing with the thickness of 4.0 nm and the lattice constant of $a = 0.737 \pm 0.09$ nm, $b = 0.569 \pm 0.15$ nm.

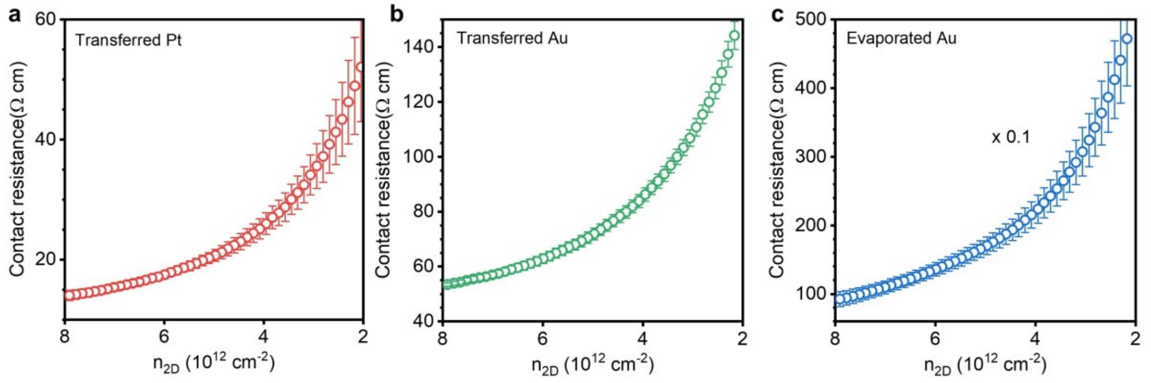

Supplementary Figure 3. Contact resistance of transferred-Pt (**a**), transferred-Au (**b**) and evaporated-Au (**c**) contact C_{10}-DNTT OTFTs as a function of carrier concentration. Among them, the transferred-Pt devices have an optimal electrical contact performance.

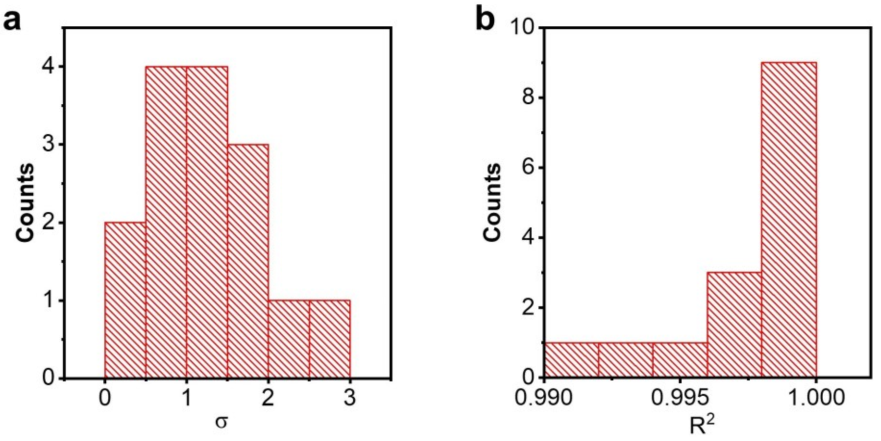

Supplementary Figure 4. Statistics of standard deviation σ (error bars in Supplementary Figure 3) and correlation coefficient R^2 and of transferred-Pt contact C_{10}-DNTT OFETs at room temperature.

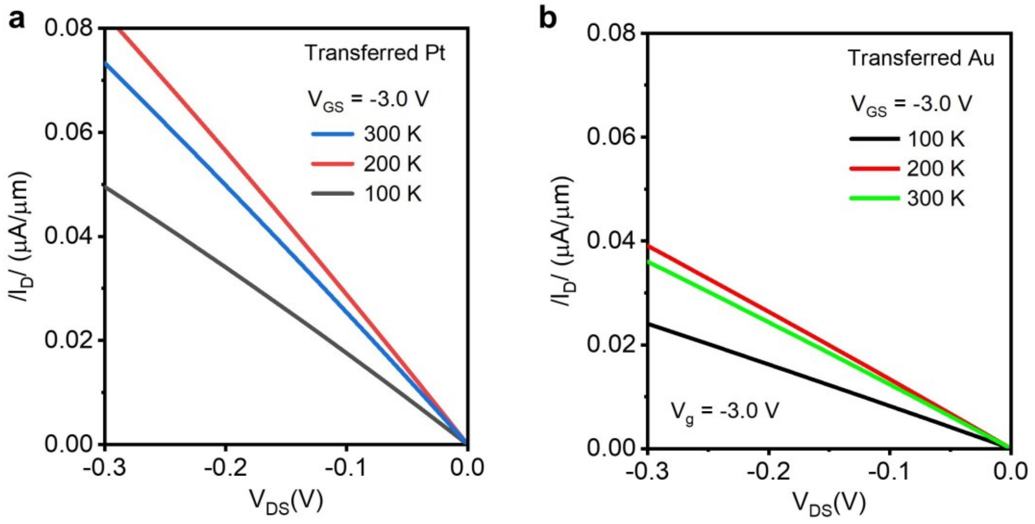

Supplementary Figure 5. (**a**) Typical $I_D \sim V_{DS}$ characteristics of transferred-Pt monolayer C_{10}-DNTT OTFTs with channel length / width of 42 / 70 μm under different temperature. (**b**) Typical $I_D \sim V_{DS}$ characteristics of transferred-Au monolayer C_{10}-DNTT OTFTs with channel length / width of 64 / 90 μm under different temperature.

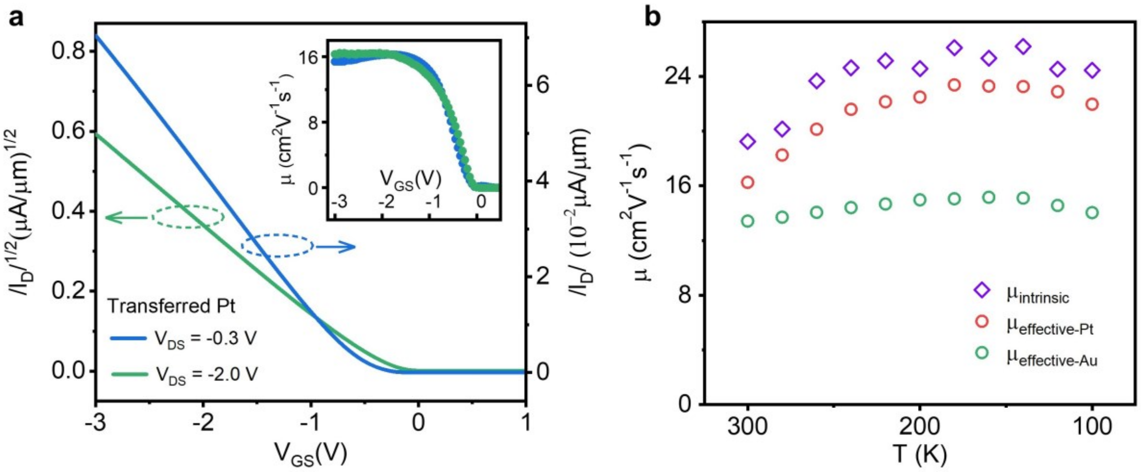

Supplementary Figure 6. (**a**) Typical $I_D \sim V_{GS}$ characteristics of transferred-Pt C_{10}-DNTT OFETs with channel length / width of 70 / 70 μm at room temperature. The excellently textbook linearity of $I_D \sim V_{GS}$ characteristics in both linear and saturation regimes is presented. Inset: two-terminal hole effective mobility in both linear (red solid circle) and saturation (red open circle) regime as a function of V_{GS}. (**b**) Two-terminal effective and intrinsic mobilities of C_{10}-DNTT OFETs as a function of temperature. These mobilities increases with temperature cooling, indicating bank like transport. The effective mobilities are nearly equivalent in both linear and saturation regimes, and almost keep constant over a large range of carrier concentration at room temperature, indicating that the channel resistance dominates the device electrical performances. The intrinsic mobility is about 10% higher than two-terminal one in transferred-Pt C_{10}-DNTT OFETs, while such ratio raises to 40% in transferred-Au devices. The average and highest two-terminal effective hole mobilities of transferred-Pt C_{10}-DNTT OFETs are 16 and 18 $cm^2V^{-1}s^{-1}$ at room temperature, nearly 20% (85%) higher than that of transferred-Au (evaporated-Au) devices.

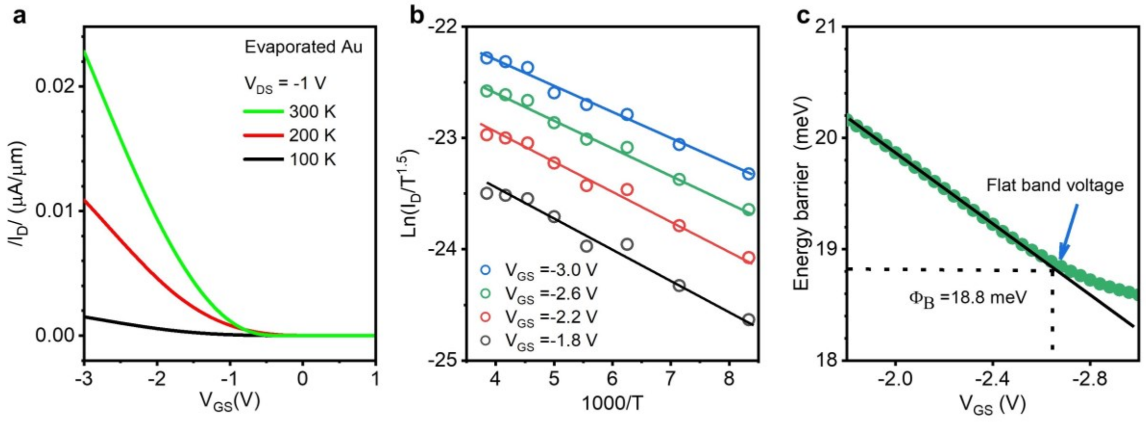

Supplementary Figure 7. (**a**) Typical $I_D \sim V_{GS}$ characteristics of evaporated-Au monolayer C_{10}-DNTT OTFT with channel length / width of 40 / 800 μm under different temperature. (**b**) Arrhenius plots of the device in **a** at $V_{DS} = -1V$. (**c**) Schottky barrier extracted from **b**. The Schottky barrier height, 18.8 meV, is extracted at the flat-band voltage.

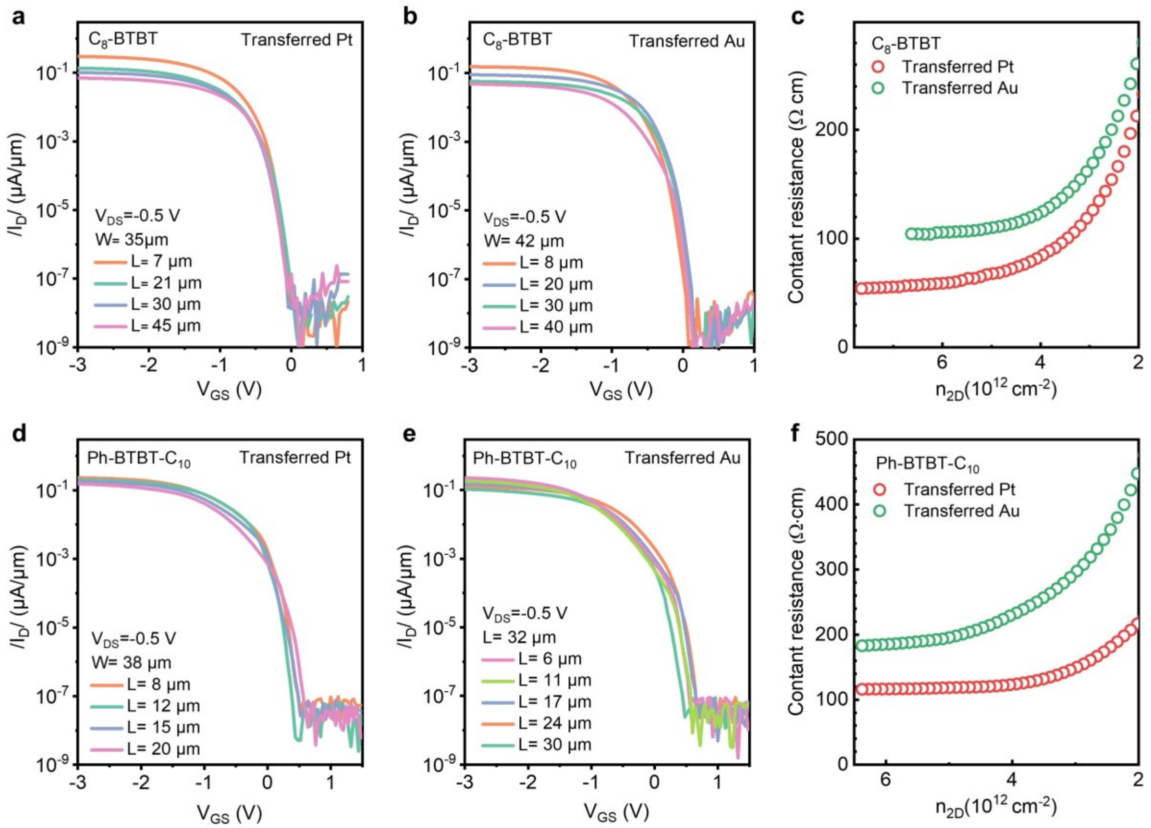

Supplementary Figure 8. (**a, b**) Room temperature $I_D \sim V_{GS}$ characteristics of a typical TLM structure of transferred-Pt (**a**) and -Au (**b**) bilayer C_8-BTBT OTFTs in the linear regime, $V_{DS} = -0.5$ V. (**c**) Contact resistance as a function of carrier concentration for transferred-Pt and -Au bilayer C_8-BTBT OTFTs, $V_{DS} = -0.5$ V. (**d, e**) Room temperature

$I_D \sim V_{GS}$ characteristics of a typical TLM structure of transferred-Pt (**d**) and -Au (**e**) bilayer Ph-BTBT-C_{10} OTFTs in the linear regime, $V_{DS} = -0.5$ V. (**f**) Contact resistance as a function of carrier concentration for transferred-Pt and -Au bilayer Ph-BTBT-C_{10} OTFTs, $V_{DS} = -0.5$ V.

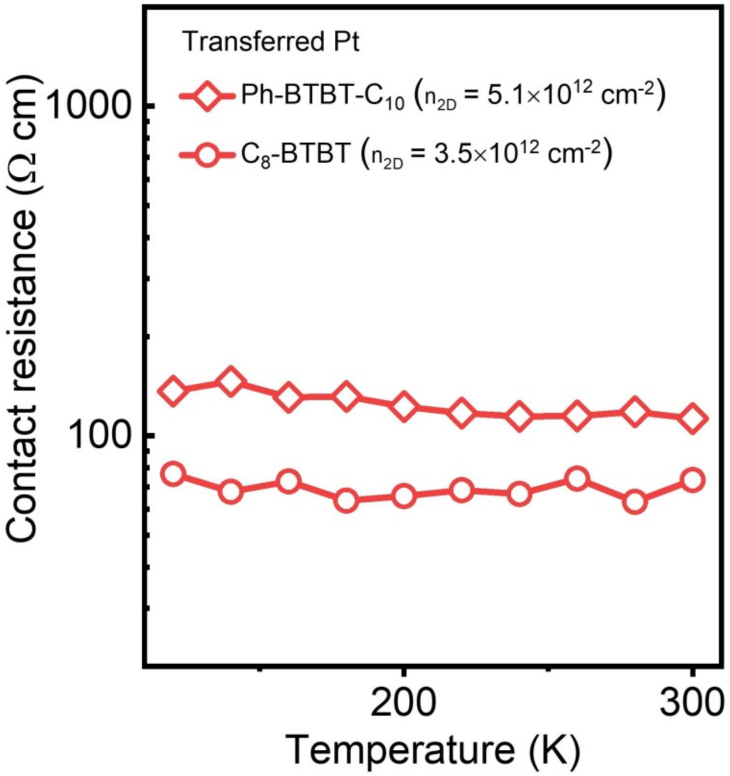

Supplementary Figure 9. The contact resistance as a function of temperature for transferred-Pt bilayer C_8-BTBT (open circle) and Ph-BTBT-C_{10} (open diamond) OTFTs.

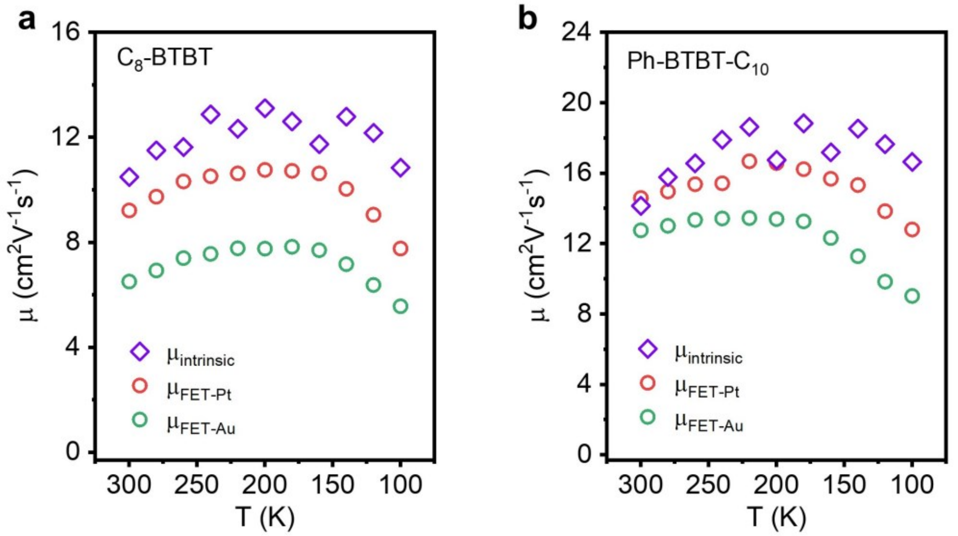

Supplementary Figure 10. (**a, b**) Two-terminal effective (circle) and intrinsic (diamond) hole mobilities of C_8-BTBT (**a**) and Ph-BTBT-C_{10} (**b**) OTFTs as a function of temperature. The two-terminal effective hole mobilities in transferred-Pt OTFTs are much closer to their intrinsic ones.

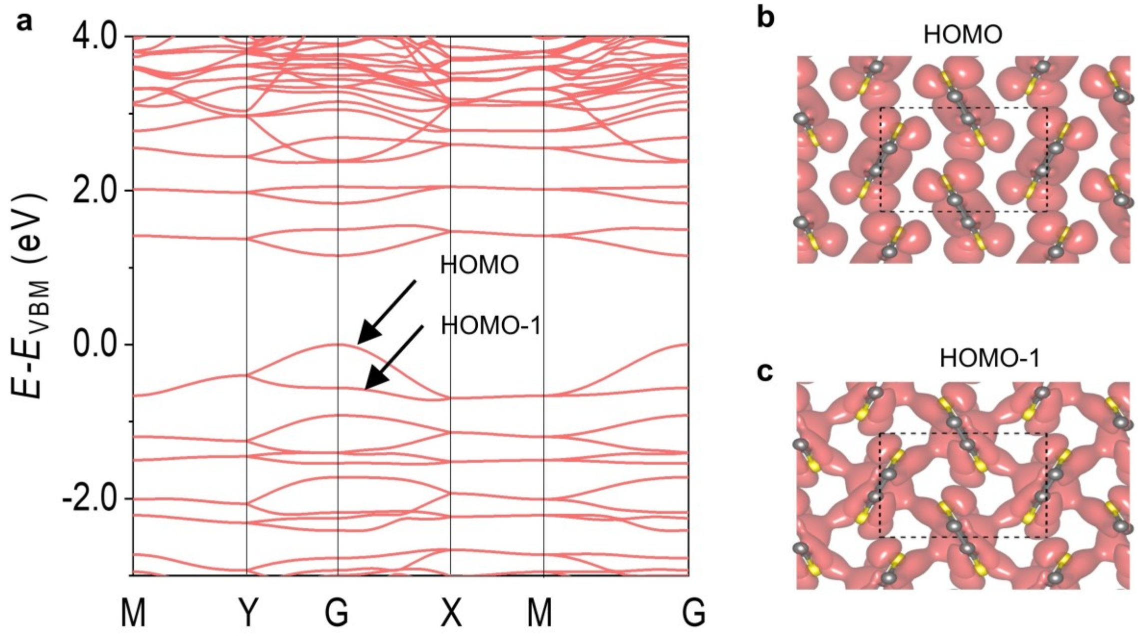

Supplementary Figure 11. (**a**) Bandstructure of monolayer C_{10}-DNTT. Two valence

states at G points of HOMO and HOMO-1 are marked. Spatial structure of wavefunctions for VBM (**b**) and VBM-1 (**c**) states at G point were illustrated in the *xy* and *yz* plane using an isosurface of 8×10^{-4} e bohr^{-3}.

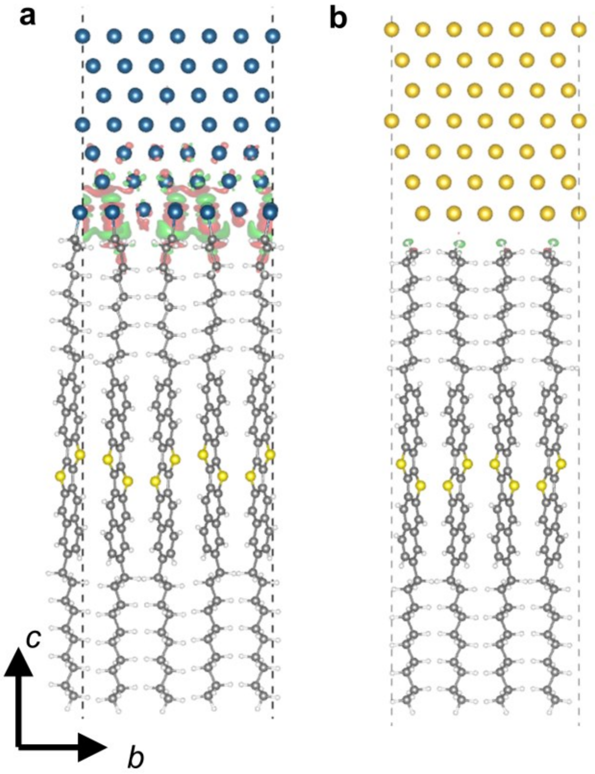

Supplementary Figure 12. (**a, b**) Geometric structures of C_{10}-DNTT/Pt (**a**) and C_{10}-DNTT/Au (**b**) contact systems. Differential charge density of the interface is shown. Pink and green colors represent charge accumulation and reduction, respectively.

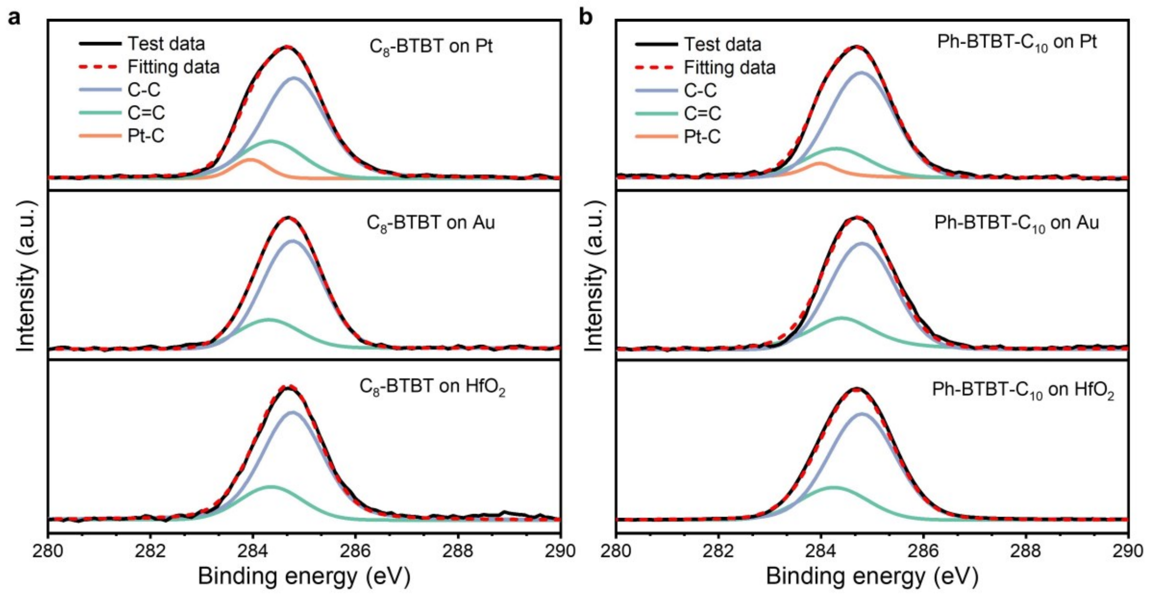

Supplementary Figure 13. XPS spectra of C_8-BTBT (**a**) and Ph-BTBT- C_{10} (**b**) on HfO_2, Au film and Pt film substrate, respectively. a.u. is arbitrary units.

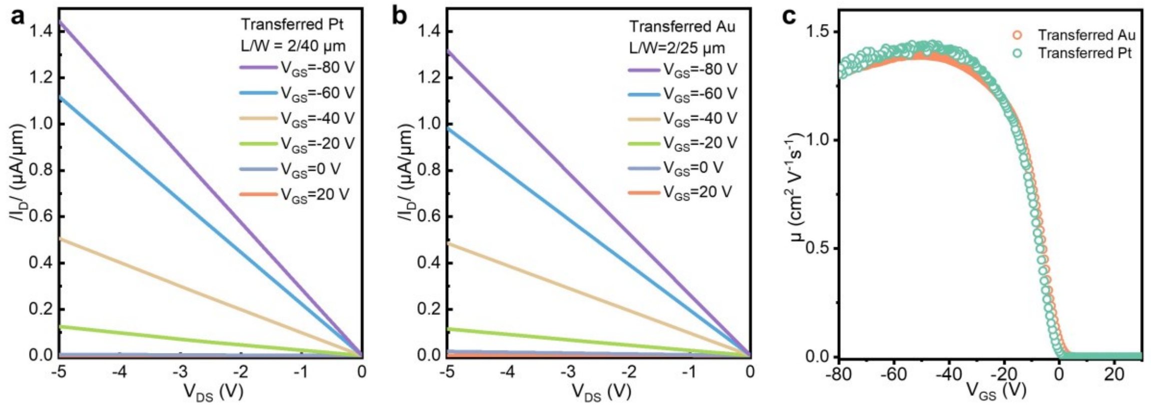

Supplementary Figure 14. The representative electrical characteristics of transferred-Pt and -Au contact pentacene OFETs with 3L single crystal thin film. The crystalline films were epitaxial grown on several nanometers BN exfoliated on 300 nm SiO_2/Si substrate by our previous study[1]. **(a)** Typical room temperature $I_D \sim V_{DS}$ characteristics of transferred-Pt contact 3L pentacene OFETs with channel length / width of 2 / 40 μm. (**b**) Typical room

temperature $I_D \sim V_{DS}$ characteristics of transferred-Au contact 3L pentacene OFETs with channel length / width of 2 / 25 μm. **(c)** Two-terminal hole effective mobility in linear regime as a function of V_{GS}, $V_{DS} = -1$ V.

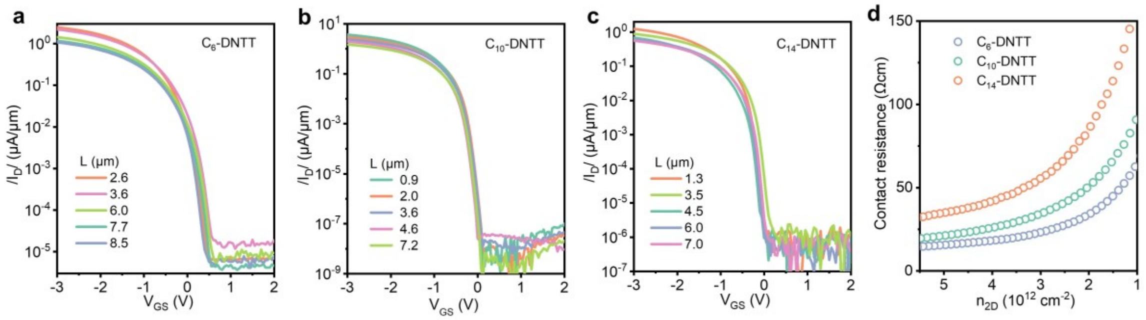

Supplementary Figure 15. (a-c) Typical room temperature $I_D \sim V_{GS}$ characteristics of a typical TLM structure of transferred-Pt C_6-DNTT (**a**), C_{10}-DNTT (**b**) and C_{14}-DNTT (**c**) OTFTs with under $V_{DS} = -1.0$ V. Each TLM structure has the same channel width. (**d**) Contact resistance as a function of carrier concentration for transferred-Pt OTFTs. The contact resistance is about 14.5 $\Omega \cdot$cm, 19.3 $\Omega \cdot$cm and 31.9 $\Omega \cdot$cm for transferred-Pt C_6-DNTT, C_{10}-DNTT and C_{14}-DNTT OTFTs at $n_{2D} = 5.5 \times 10^{12}$ cm^{-2} and $V_{DS} = -1.0$ V, respectively.

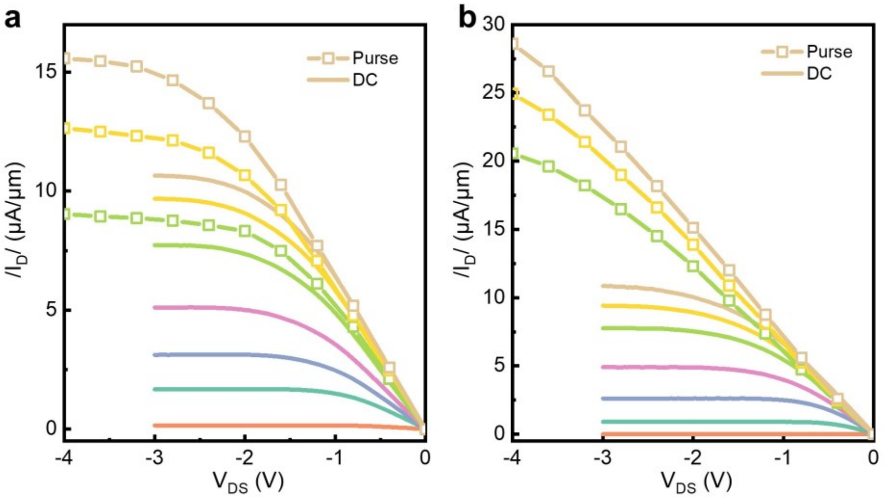

Supplementary Figure 16. The output characteristic of the C_{10}-DNTT **(a)** and C_6-DNTT **(b)** OFETs with L / W= 0.6 /10 μm. From bottom to up, V_{GS} = 0，-0.5，-1.0，-1.5，-2.0，-2.5， -3.0 V. The solid and dotted lines are the results of the DC and pulse I-V measurements, respectively.

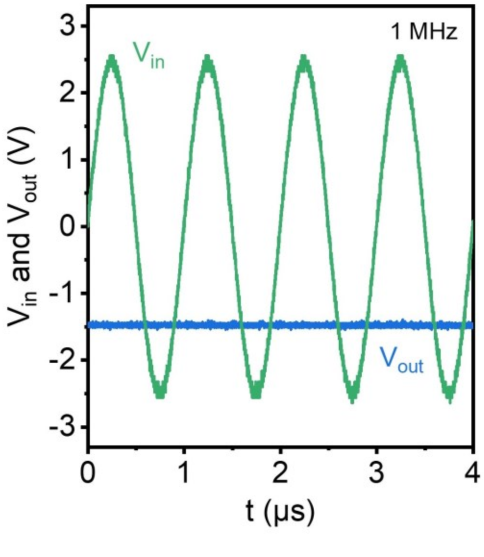

Supplementary Figure 17. The input a.c. carrier signal and output d.c. voltage as frequency of 1 MHz. The amplitude of input sinusoidal-wave voltage of 2.5 V and the load

capacitor of 82 nF.

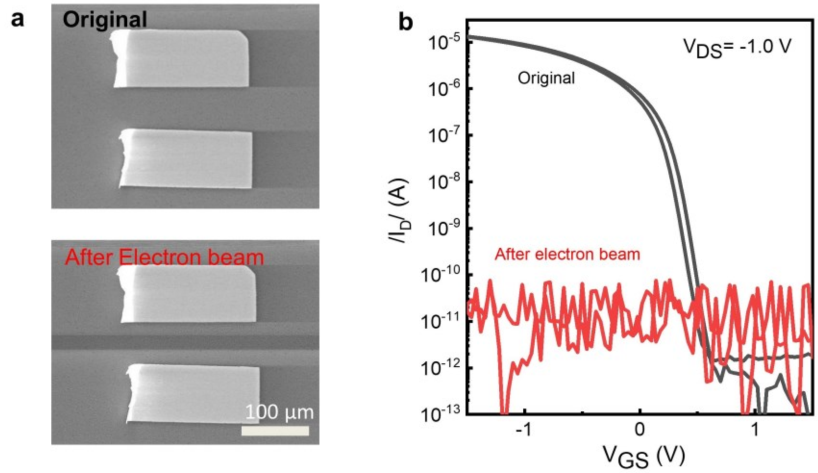

Supplementary Figure 18. (**a**) Scanning electron micrograph before and after electron-beam irradiating the channel. (**b**) the corresponding $I_D \sim V_{GS}$ characteristics tested from the device in (**a**).

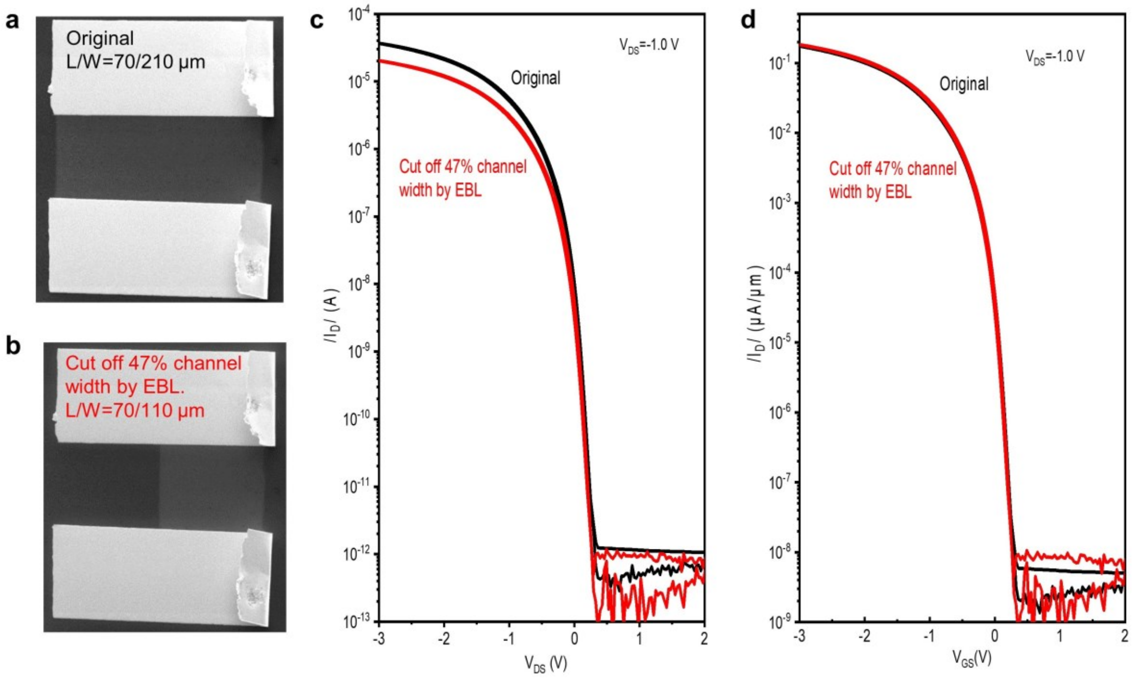

Supplementary Figure 19. The influence of fringe effect to OFET performance. (**a**) The EBL image of OFET. The channel dimension is confirmed by EBL system. The channel

width doesn't extend out the source/drain electrode width in our OFETs. (**b**) The channel width of the OFET in **a** is cut off 47% by electron beam irradiation. Comparing the current (**c**) and current density (**d**) before and after cutting off 47% channel width. The drain current decreases 45% and the current density is nearly no change (Figure R6 c and d). So, the fringe effect on drain current and effective mobility could be avoided.

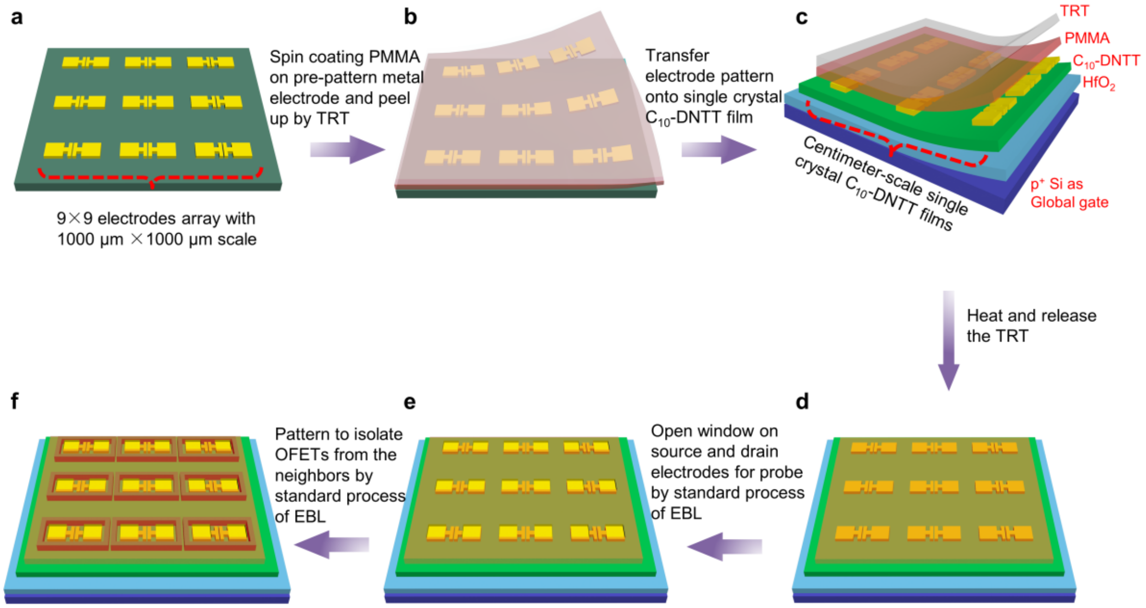

Supplementary Figure 20. The transfer process of the OFET array. (**a**) The per-pattern metal electrodes are fabricated on silicon substrate by the standard semiconductor technology. (**b**) Spin-coating PMMA onto the surface of per-pattern metal electrodes and then peel up them by thermal release tape (TRT). (**c**) Transfer the per-pattern metal electrodes onto the surface of large-area ultrathin organic semiconductor film for fabricating OTFT array. During the transfer process, we didn't need to make precisely alignment due to the scale of electrode pattern far less than the organic semiconductor film. (**d**) Release the thermal released tape at 100℃ with a very slowly temperature rising

process. (**e**) Open a window on the source and drain electrodes by the standard process of EBL for the further electrical measurement of the OFET. (**f**) Pattern the organic semiconductor film to isolate the OFETs from the neighbors by standard process of EBL, which could completely separate the adjacent two OTFTs.

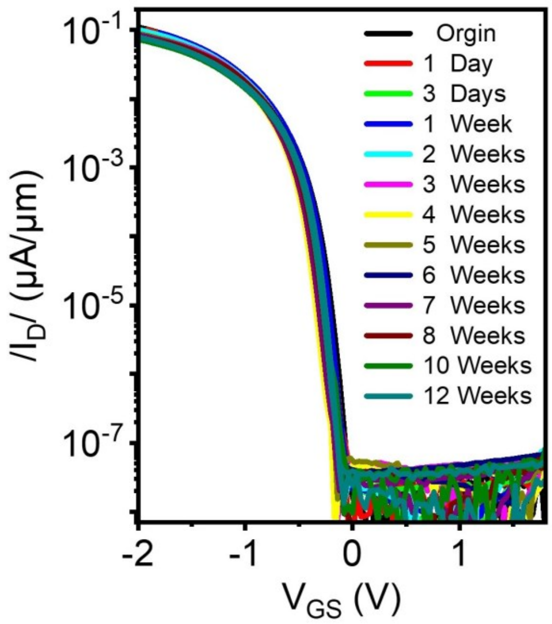

Supplementary Figure 21. Stability of the source-drain current under ambient condition over 3 months, V_{DS}=-1 V. The channel length / width is 80 / 100 µm, respectively.

Supplementary Table 1| Contact resistance comparison between typical previously reported OTFTs and average values of our devices **(partial data used in Fig.2h).**

Channel material	OFET structure	Dielectric	Strategies for Low R_CW	R_CW [Ωcm]	L_T (µm)	2D charge-carrier density [a)] [10^{13} cm^{-2}]	Ref.

**C_{10}-DNTT**	**BGTC**	**HfO_2**	**Transferred-Pt contact**	**14.0**	**0.9**	**0.79**	**This work**
**C_{10}-DNTT**	**BGTC**	**HfO_2**	**Transferred-Au contact**	**60.4**	**2.3**	**0.79**	**This work**
**C_{10}-DNTT**	**BGTC**	**HfO_2**	**Evaporated-Au contact**	**857.5**	**7.8**	**0.79**	**This work**
C_{10}-DNTT	BGTC	SiO_2	Transferred Au contact; bilayer crystalline	40	2.3	0.56	2
C_{10}-DNTT	BGTC	AlO_x/TDPA	Au contact; thin gate layer AlO_x/SAM	490	14.9	0.17	3
C_{10}-DNTT	BGTC	AlO_x/TDPA	Au contact; thin gate layer AlO_x/SAM	490	8.5	0.17	3
C_{10}-DNTT	BGTC	AlO_x/TDPA	Au contact; thin gate layer AlO_x/SAM	490	1.5	0.16	3
C_{10}-DNTT	BGTC	HfO_2	Transferred Au contact; single layer crystalline	89.9	2	0.52	4
C_{10}-DNTT	BGTC	SiO_2	Au contact; thin gate insulator	298		0.05	5
**C_8-BTBT**	**BGTC**	**HfO_2**	**Transferred-Pt contact**	**67.0**	**1.4**	**0.77**	**This work**
**C_8-BTBT**	**BGTC**	**HfO_2**	**Transferred-Au contact**	**95.2**	**2.1**	**0.77**	**This work**
C_8-BTBT	BGTC	Parylene	Au/$FeCl_3$ contact	8500		0.1	6
C_8-BTBT	TGBC	Cytop	Au/$FeCl_3$ contact	100		0.11	6
C_8-BTBT	TGTC	Cytop	Au/$FeCl_3$ contact	200		0.12	6
C_8-BTBT	BGTC	SiO_2	Transferred Au contact; single layer crystalline	400		0.1	7
**Ph-BTBT-C_{10}**	**BGTC**	**HfO_2**	**Transferred-Pt contact**	**139.2**	**2.5**	**0.64**	**This work**
**Ph-BTBT-C_{10}**	**BGTC**	**HfO_2**	**Transferred-Au contact**	**206.6**	**3.5**	**0.64**	**This work**
Ph-BTBT-C_{10}	TGTC	SiO_2	Au contact; bilayer crystalline	1200		0.14	8
Ph-BTBT-C_{10}	TGTC	SiO_2	Au contact; bilayer crystalline	2100		0.13	8
Ph-BTBT-C_{10}	BGTC	SiO_2	Au contact; bilayer crystalline	3800		0.08	8
**C_6-DNTT**	**BGTC**	**HfO_2**	**Transferred-Pt contact**	**14.5**		**0.55**	**This work**
C_6-DNTT	BGTC	SiO_2	Transferred Au contact; single layer crystalline	28		0.69	9
C_{60}	BGBC	HfO_2	Ag/OPA contact	850		0.13	10
C_{60}	BGTC	SiO_2/BCB	Al/ rhodocenedimer/ doped C_{60} contact	5500		0.02	11
C_8-DNBDT-NW	BGTC	Al_2O_3/SAM	Au/F_4-TCNQ contact; bilayer crystalline	47		0.48	12
C_9-DNBDT-NW	BGTC	Al_2O_3/SAM	Au/F_4-TCNQ contact; bilayer crystalline	60	1.8	0.3	13
C_9-DNBDT-NW	BGTC	AlO_x/Parylene-SR	Au/F_4-TCNQ contact; bilayer crystalline	50	2.5	0.16	14
CONPHINE	TGBC	SEBS-X-azide	/	2690000		0.001	15

DNTT	BGTC	AlO_x/SAM	Au contact; high-capacitance gate dielectric	200		0.14	16
DNTT	BGTC		Au/F_6-TNAP contact	110		0.4	17
DNTT	BGTC	AlO_x/TDPA	Au contact; thin gate layer AlO_x/SAM	460	8.4	0.11	3
DNTT	BGTC	AlO_x/TDPA	Au contact; thin gate layer AlO_x/SAM	460	4.2	0.11	3
DNTT	BGTC	AlO_x/TDPA	Au contact; thin gate layer AlO_x/SAM	460	0.9	0.11	3
DNTT	BGTC	AlO_x/SAM	Au/PFBT contact; thin gate insulator	600	10	0.14	18
DPh-DNTT	BGBC	AlO_x/SAM	Au/PFBT contact; thin gate insulator	12		0.66	19
DPh-DNTT	BGBC	Al_2O_3/HC_{14}-PA	Au/PFBT contact; thin gate insulator	29	1.1	0.8	20
DPh-DNTT	BGBC	Al_2O_3/HC_{14}-PA	Au/PFBT contact; thin gate insulator	10	1.1	0.64	21
DPh-DNTT	BGBC	Al_2O_3/HC_{14}-PA	Au/PFBT contact; thin gate insulator	35		0.7	21
DPh-DNTT	BGTC	Al_2O_3/HC_{14}-PA	Au contact; thin gate insulator	56	2.4	0.8	20
DPh-DNTT	BGTC	AlO_x/TDPA	Au contact; thin gate layer AlO_x/SAM	240	7.3	0.15	3
DPh-DNTT	BGTC	AlO_x/TDPA	Au contact; thin gate layer AlO_x/SAM	240	4.1	0.13	3
DPh-DNTT	BGTC	AlO_x/TDPA	Au contact; thin gate layer AlO_x/SAM	240	1.1	0.13	3
DPPT-TT/SEBS	BGBC	SiO_2	/	97500		0.01	22
IDTBT	BGBC	Cytop	/	80		0.4	23
IDTBT	TGBC	PMMA	/	2300000		0.03	24
I-PCDTPT	BGBC	SiO_2	/	14700		0.19	25
N-PCDTPT	BGBC	SiO_2	/	65200		0.01	25
P3HT	TGBC	Ion gel	High charge-carrier density from electrolyte gate insulator	1		16	26
P3HT	TGBC	Ion gel	High charge-carrier density from electrolyte gate insulator	15			27
PCDTPT	BGBC	SiO_2		30900		0.07	25
PCDTPT	BGBC	SiO_2	/	49200		0.14	28
PDIF-CN_2	BGTC	SiO_2	Au contact; thin gate insulator	3620		0.05	5
PDPP	TGBC	Ionic liquid	High charge-carrier density from electrolyte gate insulator	2.7		2.4	29

Pentacene	BGBC	SiO_2	Au contact; UV/ozone treated	80		0.4	30
Pentacene	BGTC	AlO_x/TDPA	Au contact; thin gate layer AlO_x/SAM	1400	10.4	0.12	3
Pentacene	BGTC	AlO_x/TDPA	Au contact; thin gate layer AlO_x/SAM	1400	5	0.12	3
Pentacene	BGTC	AlO_x/TDPA	Au contact; thin gate layer AlO_x/SAM	1400	2.1	0.13	3
Pentacene	BGBC	HfO_2	Ag/OPA contact	700		0.07	10
PTCDI-C_{13}	BGBC	SiO_2	/	60000		0.02	31
TIPS- Pentacene	BGTC	SiO_2	Au contact; thin gate insulator	2240		0.04	5
TIPS-pentacene /PTAA	TGBC	Cytop	/	351000		0.001	32
TIPS-pentacene /PTAA	TGBC	Cytop	Au/ PFBT contact; bilayer crystalline	65000		0.01	32
TIPS-pentacene /PTAA	TGBC	Cytop	Au/ Mo(tfd)$_3$ contact; bilayer crystalline	13000		0.01	32
TIPS-pentacene/ PTAA	TGBC	Cytop	Au/ MoO_3 contact; bilayer crystalline	11000		0.01	32

Supplementary Table 2| Key performance metrics comparation between typical previously reported OTFTs and best values of our devices **(partial data used in Fig. 2g)**

Channel material	OFET structure	Dielectric	S/D Contact	μ [cm^2 V^{-1} s^{-1}]	V_{dd} [V]	V_{th} [V]	SS [mV/dec]	Ref.
**C_{10}-DNTT**	**BGTC**	**HfO_2**	**Transferred-Pt**	**18.01**	**-3**	**-0.09**	**59.8 (300K)**	**This work**
**C_{10}-DNTT**	**BGTC**	**HfO_2**	**Transferred-Au**	**15.4**	**-3**	**-0.15**	**60 (300K)**	**This work**
**C_{10}-DNTT**	**BGTC**	**HfO_2**	**Evaporated-Au**	**4.32**	**-3**	**-0.6**	**83.7 (300K)**	**This work**
C_{10}-DNTT	BGTC	SiO_2	Au	12.5	-80	2.1	330	2
C_{10}-DNTT	BGTC	AlO_x/TDPA	Au	5.3	-3	-0.62	81	3
C_{10}-DNTT	BGTC	AlO_x/TDPA	Au	0.38	-3	-0.73	121	3
C_{10}-DNTT	BGTC	AlO_x/TDPA	Au	2.1	-3	-0.64	102	3
C_{10}-DNTT	BGTC	AlOx/SAM	Au/NDP-9/DNTT/NDP-9	4.3	-3	-0.4	68	33
C_{10}-DNTT	BGTC	PSQ	Au	27.9	-3	-1.42	62	34
**C_8-BTBT**	**BGTC**	**HfO_2**	**Transferred-Pt**	**10.1**	**-2**	**-0.2**	**60.8 (300K)**	**This work**
**C_8-BTBT**	**BGTC**	**HfO2**	**Transferred-Au**	**7.6**	**-2**	**-0.2**	**61.3 (300K)**	**This work**
C_8-BTBT	BGTC	Parylene	Au/$FeCl_3$	7	-40	-10	210	6

C_8-BTBT	TGBC	Cytop	Au/$FeCl_3$	5.5	-40	-0.81	240	6
C_8-BTBT	TGBC	Cytop	Au/$FeCl_3$	2.2	-40	-2.2	700	6
C_8-BTBT	TGTC	Cytop	Au/$FeCl_3$	5.7	-40	1.1	230	6
C_8-BTBT	BGTC	SiO_2	Au	13	-20	-4	400	7
**Ph-BTBT-C_{10}**	**BGTC**	**HfO_2**	**Transferred-Pt**	**14.1**	**-2**	**-0.15**	**60 (300K)**	**This work**
**Ph-BTBT-C_{10}**	**BGTC**	**HfO_2**	**Transferred-Au**	**12.9**	**-2**	**-0.17**	**63.2 (300K)**	**This work**
Ph-BTBT-C_{10}	BGBC	SiO_2	Au	11.2	-50	-3	780	8
Ph-BTBT-C_{10}	BGBC	SiO_2	Au/PFBT	4.9	-2	0.12	79	35
C_{60}	BGBC	HfO_2/OPA	Ag/OPA	0.6	-3	1.6	75	10
C_{60}	BGBC	HfO_2/OPA	Ag/OPA	3.7	-3	-0.6	95	10
C_{60}	BGTC	pV3D3	Al/Ga	1.32	2	0.64	173	36
C_8-BTBT:PS	BGBC	PVC	Ag	5	-3	-0.01	61	37
C_8-BTBT:PS	BGTC	AlO_x	Ag/F_4-TCNQ	8.7	-4	-1.5	71	38
C_9-DNBDT-NW	BGTC	Parylene	Au/F_4-TCNQ	10.1	-30	-5	160	39
DNTT	BGTC	AlO_x/TDPA	Au	3.7	-3	-1.47	86	3
DPh-BTBT	BGBC	TiO_2/HC14-PA	Au	0.18	-1	-0.45	59 (293K)	19
DPh-DNTT	BGBC	Al_2O_3/HC14-PA	Au	4.9	-3	-1	62	20
DPh-DNTT	BGBC	Al_2O_3/HC14-PA	Au/PFBT	4.3	-3	-1.2	59 (292K)	21
DPh-DNTT	BGTC	Al_2O_3/HC14-PA	Au/PFBT	5.7	-3	-1	92	20
DPh-DNTT	BGTC	AlO_x/TDPA	Au	5.9	-3	-1.38	84	3
DPh-DNTT	BGTC	AlO_x/TDPA	Au	0.68	-3	-1.17	101	3
PDVT-8	BGBC	AlO_x/OTS	Au/PFBT	2.4	-2	-0.24	65	40
Pentacene	BGTC	CEP/ Al_2O_3	Au	6.5	-3	-1.46	62	41
Pentacene	BGTC	AlO_x/TDPA	Au	1.2	-3	-1.28	138	3
Pentacene	BGTC	Copolymer	Au	5.6	-3	-0.5	220	42
Ph-BTNT-C_n	BGBC	Cytop/ SiO_2	Au	5.5	-2	-2	63	43
PIDT-BT:TCNQ	TGBC	PAA:PEG	Au	0.31	-0.7	-0.28	62	44
PTDPPTFT$_4$	BGTC	PVP	Au	0.129	-5	-0.66	110	45
Rubrene	BGBC	Cytop	Au	13.9	-10	-0.23	65	46
TES-ADT	BGTC	PS brush/ AlOx	Au	1.26	-1	-0.24	85	47
TIPS-pentacene: PS	BGBC	Terpolymer	Ag/PFBT	0.4	-5		64	48
TIPS-pentacene	BGTC	PVP	Au	0.171	-5	-1.34	110	45

μ, V_{dd}, V_{th}, SS are two-terminal field effect effective mobility, saturation voltage, threshold voltage and subthreshold swing, respectively. Compared with SiO2, polymer or other dielectrics, a thinner HfO2 substrate is beneficial to grow high quality ultrathin

crystalline thin-films due to lower surface energy and roughness, to gate higher carrier concentration that could effectively lower the contact resistance and driving voltage due to a higher capacitance. In addition, the alkyl chains of organic molecules that could be regarded as self-assembled monolayers can weaken the high polarizability of high-k dielectric[49]. These may be another important reason that our OTFTs deliver the excellent electrical properties.

Supplementary Table 3| Saturation current density comparison between typical previously reported OTFTs and our devices **(partial data used in Fig. 4c).**

Channel material	Dielectric	S/D Contact	L [μm]	E [V/μm]	Saturation current density [μA/μm]	Ref.
**C_{10}-DNTT**	**HfO_2**	**Pt**	0.6	**3.8**	**15.8**	**This Work**
C_{10}-DNTT	SiO_2	Au	8	7.5	4.2	2
C_{10}-DNTT	SiO_2	Au/F_6TNAP	60	1	1.8	17
C_{10}-DNTT	AlO_x/TDPA	Au	100	0.03	0.1	3
C_{10}-DNTT	AlO_x/TDPA	Au	1	3	0.75	3
C_{10}-DNTT	SiO_2	Au	76	1.1	5	50
C_{10}-DNTT	SiO_2	Au	100	0.8	2	5
C_{10}-DNTT	SiO_2	Au	80	1	1.7	51
C_{10}-DNTT	SiO_2	Au	140	0.43	0.93	52
C_{10}-DNTT	AlO_x/SAM	Au/NDP-9/DNTT/NDP-9	30	0.1	0.12	33
**C_8- BTBT**	**HfO_2**	**Pt**	1	**2**	**1.1**	**This Work**
C_8- BTBT	Cytop	Au/$FeCl_3$	50	0.8	0.08	6
C_8- BTBT	Parylene C	Au	100	0.5	0.35	53
C_8- BTBT	SiO_2	Au	200	0.4	0.55	54
C_8- BTBT	BN	Au/Graphene	10	0.4	0.15	55
C_8- BTBT	SiO_2	Au	100	0.2	0.53	7
C_8- BTBT	SiO_2	Au	100	0.033	0.2	56
**Ph-BTBT-C_{10}**	**HfO_2**	**Pt**	0.6	**3.3**	2.0	**This Work**
Ph-BTBT-C_{10}	SiO_2	Au	190	0.26	0.25	57
Ph-BTBT-C_{10}	SiO_2	Au	100	1	0.18	8
Ph-BTBT-C_{10}	SiO_2	Au	100	2	0.9	8

Ph-BTBT-C_{10}	SiO_2	Au	100	0.5	1.1	8
Ph-BTBT-C_{10}	SiO_2/PS	Au/PFBT	70	0.029	0.003	35
Ph-BTBT-C_{10}	SiO_2	Au	100	0.5	0.2	58
**C_6-DNTT**	**HfO_2**	**Pt**	0.6	6.67	**28.6**	**This Work**
C_6-DNTT	SiO_2	Au	5.3	15.1	19	9
C_{12}-BTBT	SiO_2	Ag/F_4-TCNQ	5	12	6	59
C_{12}-BTBT	PhTS/ SiO_2	$FeCl_3$/Au	100	0.4	0.52	60
C_{12}-BTBT	PMMA/SiO_2	Au	120	0.67	0.011	61
C_{60}	SiO_2/BCB	Al/ rhodocenedimer	25	0.96	0.17	11
C_{60}	SiO_2/BCB	Al/ rhodocenedimer	200	0.12	0.03	11
C_{60}	pV3D3	Al/Ga	200	0.15	0.034	36
C_8-BTBT/PS	PVC	Ag	40	0.075	3.5×10^{-4}	37
C_8-BTBT/PS	PVP/HDA	Ag	100	0.05	0.34	62
C_8-BTBT/PS	Cytop/ Parylene	Au	100	0.08	0.1	63
C_8-DNBDT-NW	Al_2O_3/SAM	Au/F_4-TCNQ	3	3.3	1.6	12
C_8-DNBDT-NW	SiO_2	Au/F_4-TCNQ	32	0.94	0.14	12
C_8-DNBDT-NW	SiO_2	Au/F_4-TCNQ	33	0.91	0.17	12
C_8-DNBDT-NW	β-PTS SiO_2	Au	200	0.1	0.37	64
C_9-DNBDT-NW	Al_2O_3/SAM	Au/F_4-TCNQ	6	1.2	2	13
C_9-DNBDT-NW	AlOX/Parylene-SR	Au/F_4-TCNQ	50	0.3	0.9	14
C_9-DNBDT-NW	AlOX/Parylene-SR	Au/F_4-TCNQ	1.5	10	2.6	14
C_9-DNBDT-NW	Parylene	Au/F_4-TCNQ	100	0.3	0.96	39
CONPHINE	SEBS-X-azide	CNT	70	0.43	0.01	15
DNTT	AlOx/SAM	Au	4	0.75	0.14	16
DNTT	AlOx/SAM	Au	8	0.38	0.08	16
DNTT	AlOx/SAM	Au	12	0.25	0.06	16
DNTT	AlOx/TDPA	Au	100	0.03	0.03	3
DNTT	AlOx/TDPA	Au	1	3	0.35	3
DNTT	Parylene	Au	50	0.16	0.04	65
DNTT	Polyimide	Au	50	0.4	0.052	65
DNTT	SiO_2/OTS	Ag	100	0.5	0.25	66
DNTT	AlOx/	Au	50	0.06	0.001	67
DPh-DNTT	Al_2O_3/HC_{14}-PA	Au/PFBT	1	3	2.4	20
DPh-DNTT	Al_2O_3/HC_{14}-PA	Au	1	3	1.2	20

DPh-DNTT	Al_2O_3/HC_{14}-PA	Au/PFBT	0.6	5	6.5	21
DPh-DNTT	AlO_x/TDPA	Au	100	0.03	0.08	3
DPh-DNTT	AlO_x/TDPA	Au	1	3	0.95	3
DPh-DNTT	TiO_x /SAM	Au	25	0.025	0.0038	68
$DPPPHF_4$	SiO_2	Au	200	0.4	0.02	69
DPPT-TT/SEBS	SiO_2	Au	200	0.4	0.07	22
DPPT-TT/SEBS	SEBS	CNT	200	0.15	0.003	22
F_{16}CuPc	SAM/AlO_x	Au	50	0.04	2.0×10^{-4}	70
F_{16}CuPc	SiO_2/C_8-PFTS	Ag	100	0.5	7.5×10^{-4}	66
IDTBT	Cytop	Au	20	3	0.5	23
IDTBT	PMMA	Au	100	0.5	0.0017	24
P3HT	PS brush/ SiO_2	Au	100	0.8	0.005	47
PCDTPT	SiO_2	Au/Ni	160	0.5	0.9	25
PCDTPT	SiO_2	Au/Ni	160	0.5	0.85	25
PCDTPT	SiO_2	Au/Ni	80	1	0.8	28
PCDTPT	SiO_2	Au/Ni	160	0.5	1	28
PDBPyBT	Cytop	Au	30	2.7	0.35	71
PDIF-CN_2	SiO_2	Au	100	0.6	0.05	5
pentacene	AlO_x/TDPA	Au	100	0.03	0.01	3
pentacene	AlO_x/TDPA	Au	1	3	0.3	3
pentacene	HfO_2/OPA	Ag/OPA	20	0.15	0.037	10
PhC_2−BQQDI	AL-X601/ AlO_x	Au	6	3.3	0.08	72
PTCDI–C_{13}	PyB/ SiO_2	Au	50	2	0.5	73
PTCDI–C_{13}	PS brush/ SiO_2	Au	100	0.4	0.03	47
PTCDI–C_{13}	SiO_2	Au/PEDOT	150	0.4	0.0026	31
$PTDPPTFT_4$	PVP	Au	50	0.1	0.01	45
$PTDPPTFT_4$	SiO_2	Au	50	1.2	0.08	74
$PTDPPTFT_4$	PVP	Au	50	2	0.3	75
TES-ADT	PS brush/ SiO_2	Au	100	0.4	0.01	47
TIPS- Pentacene	SiO_2	Au	5	8	1.6	76
TIPS- Pentacene	PVP	Au	50	0.1	0.02	45
TIPS- Pentacene	SiO_2	Au	10	6	0.03	5

Supplementary Table 4| Summary of normalized rectification frequency (f_R/V_0) of Organic diodes

Semiconductor material	Fabrication techniques	Structure	μ[a)] [cm^2 V^{-1} s^{-1}]	V_0[b)] [V]	-3dB Frequency [c)] [MHz]	f_R/V_0 [MHz V^{-1}]	Year	Ref.
**Au/Pt/C_{10}-DNTT/Pt/Au**	**Van der Waals contact**	**Co-planar**	**18**	**2.5**	**64**	**25.6**	**2023**	**This work**
Au/F_4-TCNQ/C_8-DNBDT-NW/F_4-TCNQ/Au	Thermal evaporation	Co-planar	13	8	29	3.6	2018	12
Au/F_4-TCNQ/C_9-DNBDT-NW/F_4-TCNQ/Au	Thermal evaporation	Co-planar	11	5	78	15.6	2020	14
Al/DNTT/Al	Thermal evaporation	Co-planar	0.44	15	20 (63%)	1.3	2015	77
Au/PEDOT:PSS /pentacene/Al	Thermal evaporation	sandwich	5.2×10^{-5}	10	13.56	6.78	2005	78
Au/pentacene/PEDOT:PSS/Al	Deposition	sandwich	0.15	18	14 (61%)	0.78	2006	79
Au/CuPc/Al	Spin-coating	sandwich	4×10^{-3}	5	14 (40%)	2.8	2007	80
Cu/CuTCNQ/pentacene/Al	Thermal evaporation	sandwich	1.5×10^{-3}	5	13.56 (40%)	2.71	2010	81
IZO/PEDOT:PSS/PQT-12/Al	Spin-coating	sandwich	0.7	10	13.56 (40%)	1.36	2011	82
Ag/PTAA/Ag	Thermal evaporation	sandwich		10	13.56 (51%)	1.36	2014	83
Ag/PEI/P(NDI2OD-T2)/Ag	Thermal evaporation	sandwich	1.0	5	13.56 (90%)	2.71	2020	84
Au/F_{16}CoPc/CuPc/Au	Deposition	sandwich		2.5	10	4	2020	85
Au/PFBT/P3HT/Al	Thermal evaporation	sandwich	5.5×10^{-2}	2	6.2	3.1	2021	86
Au/pentacene/Al	Thermal evaporation	sandwich	0.15	15	433	28.9	2008	87
Au/PFBT/pentacene/Al	Thermal evaporation	sandwich	0.11	10	1240	124	2016	88
Al/WO_3-HMDS/C_{60}/BCP/Al	Thermal evaporation	sandwich	0.42	2	700	350	2011	89

a) μ refers to the maximum effective mobility reported.
b) V_0 refers to the amplitude of the input ac voltage
c) When the attenuation of V_{out} is not exactly -3dB (~71%), the remaining proportion of V_{out} corresponding to this frequency will be marked in parentheses.

Supplementary References

1. Zhang, Y. *et al.* Probing carrier transport and structure-property relationship of highly ordered organic semiconductors at the two-dimensional limit. *Physical review letters* **116**, 016602 (2016).
2. Peng, B. *et al.* Crystallized monolayer semiconductor for ohmic contact resistance, high intrinsic gain, and high current density. *Advanced Materials* **32**, 2002281 (2020).
3. Kraft, U. *et al.* Detailed analysis and contact properties of low-voltage organic thin-film transistors based on dinaphtho [2, 3-b: 2′, 3′-f] thieno [3, 2-b] thiophene (DNTT) and its didecyl and diphenyl derivatives. *Organic Electronics* **35**, 33-40 (2016).
4. Chen, M., Peng, B., Sporea, R. A., Podzorov, V. & Chan, P. K. L. The Origin of Low Contact Resistance in Monolayer Organic Field-Effect Transistors with van der Waals Electrodes. *Small Science*, 2100115 (2022).
5. Pei, K., Chen, M., Zhou, Z., Li, H. & Chan, P. K. L. Overestimation of carrier mobility in organic thin film transistors due to unaccounted fringe currents. *ACS Applied Electronic Materials* **1**, 379-388 (2019).
6. Darmawan, P. *et al.* Optimal Structure for High-Performance and Low-Contact-Resistance Organic Field-Effect Transistors Using Contact-Doped Coplanar and Pseudo-Staggered Device Architectures. *Advanced Functional Materials* **22**, 4577-4583 (2012).
7. Wang, Q. *et al.* 2D Single-Crystalline Molecular Semiconductors with Precise Layer Definition Achieved by Floating-Coffee-Ring-Driven Assembly. *Advanced Functional*

Materials **26**, 3191-3198 (2016).

8. Iino, H., Usui, T. & Hanna, J.-i. Liquid crystals for organic thin-film transistors. *Nature communications* **6**, 6828, 1-8 (2015).
9. Peng, B., He, Z., Chen, M. & Chan, P. K. L. Ultrahigh On-Current Density of Organic Field-Effect Transistors Facilitated by Molecular Monolayer Crystals. *Advanced Functional Materials*, 2202632 (2022).
10. Acton, O. *et al.* Simultaneous modification of bottom-contact electrode and dielectric surfaces for organic thin-film transistors through single-component spin-cast monolayers. *Advanced Functional Materials* **21**, 1476-1488 (2011).
11. Singh, S. *et al.* Reduction of contact resistance by selective contact doping in fullerene n-channel organic field-effect transistors. *Applied Physics Letters* **102**, 63 (2013).
12. Yamamura, A. *et al.* Wafer-scale, layer-controlled organic single crystals for high-speed circuit operation. *Science advances* **4**, eaao5758 (2018).
13. Sawada, T. *et al.* Correlation between the static and dynamic responses of organic single-crystal field-effect transistors. *Nature communications* **11**, 1-8 (2020).
14. Yamamura, A. *et al.* High-Speed Organic Single-Crystal Transistor Responding to Very High Frequency Band. *Advanced Functional Materials* **30**, 1909501 (2020).
15. Wang, S. *et al.* Skin electronics from scalable fabrication of an intrinsically stretchable transistor array. *Nature* **555**, 83-88 (2018).
16. Elsobky, M. *et al.* A digital library for a flexible low-voltage organic thin-film transistor technology. *Organic Electronics* **50**, 491-498 (2017).

17. Matsumoto, T., Ou-Yang, W., Miyake, K., Uemura, T. & Takeya, J. Study of contact resistance of high-mobility organic transistors through comparisons. *Organic Electronics* **14**, 2590-2595 (2013).

18. Ante, F. *et al.* Contact resistance and megahertz operation of aggressively scaled organic transistors. *small* **8**, 73-79 (2012).

19. Geiger, M. *et al.* Subthreshold Swing of 59 mV decade− 1 in Nanoscale Flexible Ultralow-Voltage Organic Transistors. *Advanced Electronic Materials* **8**, 2101215 (2022).

20. Borchert, J. W. *et al.* Small contact resistance and high-frequency operation of flexible low-voltage inverted coplanar organic transistors. *Nature communications* **10**, 1-11 (2019).

21. Borchert, J. W. *et al.* Flexible low-voltage high-frequency organic thin-film transistors. *Science advances* **6**, eaaz5156 (2020).

22. Xu, J. *et al.* Highly stretchable polymer semiconductor films through the nanoconfinement effect. *Science* **355**, 59-64 (2017).

23. Venkateshvaran, D. *et al.* Approaching disorder-free transport in high-mobility conjugated polymers. *Nature* **515**, 384-388 (2014).

24. Zou, J. *et al.* Flexible Organic Thin-Film Transistors With High Mechanical Stability on Polyimide Substrate by Chemically Plated Silver Electrodes. *IEEE Transactions on Electron Devices* **68**, 5120-5126 (2021).

25. Lee, B. H., Bazan, G. C. & Heeger, A. J. Doping-Induced Carrier Density Modulation

in Polymer Field-Effect Transistors. *Advanced Materials* **28**, 57-62 (2016).

26. Braga, D., Ha, M., Xie, W. & Frisbie, C. D. Ultralow contact resistance in electrolyte-gated organic thin film transistors. *Applied Physics Letters* **97**, 245 (2010).
27. Thiburce, Q., Giovannitti, A., McCulloch, I. & Campbell, A. J. Nanoscale ion-doped polymer transistors. *Nano letters* **19**, 1712-1718 (2019).
28. Luo, C. *et al.* General strategy for self-assembly of highly oriented nanocrystalline semiconducting polymers with high mobility. *Nano letters* **14**, 2764-2771 (2014).
29. Lenz, J., Del Giudice, F., Geisenhof, F. R., Winterer, F. & Weitz, R. T. Vertical, electrolyte-gated organic transistors show continuous operation in the MA cm− 2 regime and artificial synaptic behaviour. *Nature nanotechnology* **14**, 579-585 (2019).
30. Stadlober, B. *et al.* Orders-of-magnitude reduction of the contact resistance in short-channel hot embossed organic thin film transistors by oxidative treatment of Au-electrodes. *Advanced functional materials* **17**, 2687-2692 (2007).
31. Hong, K. *et al.* Photopatternable, highly conductive and low work function polymer electrodes for high-performance n-type bottom contact organic transistors. *Organic Electronics* **12**, 516-519 (2011).
32. Choi, S. *et al.* A study on reducing contact resistance in solution-processed organic field-effect transistors. *ACS applied materials & interfaces* **8**, 24744-24752 (2016).
33. Zschieschang, U. *et al.* Flexible low-voltage organic thin-film transistors and circuits based on C 10-DNTT. *Journal of Materials Chemistry* **22**, 4273-4277 (2012).
34. Ye, H. *et al.* The Hidden Potential of Polysilsesquioxane for High-k: Analysis of the

Origin of its Dielectric Nature and Practical Low-Voltage-Operating Applications beyond the Unit Device. *Advanced Functional Materials* **32**, 2104030 (2022).

35. Kunii, M., Iino, H. & Hanna, J.-I. Solution-Processed, Low-Voltage Polycrystalline Organic Field-Effect Transistor Fabricated Using Highly Ordered Liquid Crystal With Low-$ k $ Gate Dielectric. *IEEE Electron Device Letters* **37**, 486-488 (2016).
36. Moon, H. *et al.* Synthesis of ultrathin polymer insulating layers by initiated chemical vapour deposition for low-power soft electronics. *Nature materials* **14**, 628-635 (2015).
37. Jiang, C. *et al.* Printed subthreshold organic transistors operating at high gain and ultralow power. *Science* **363**, 719-723 (2019).
38. Duan, S. *et al.* Solution-Processed Centimeter-Scale Highly Aligned Organic Crystalline Arrays for High-Performance Organic Field-Effect Transistors. *Advanced Materials* **32**, 1908388 (2020).
39. Makita, T. *et al.* High-performance, semiconducting membrane composed of ultrathin, single-crystal organic semiconductors. *Proceedings of the National Academy of Sciences* **117**, 80-85 (2020).
40. Kim, S. H. *et al.* High performance ink-jet printed diketopyrrolopyrrole-based copolymer thin-film transistors using a solution-processed aluminium oxide dielectric on a flexible substrate. *Journal of Materials Chemistry C* **1**, 2408-2411 (2013).
41. Xu, W., Wang, F. & Rhee, S.-W. Quasi-ordering in spontaneously associated surface dipoles: an intrinsic interfacial factor for high-k polymer insulated organic field-effect transistors. *Journal of Materials Chemistry* **22**, 1482-1488 (2012).

42. Ji, D. *et al.* Copolymer dielectrics with balanced chain-packing density and surface polarity for high-performance flexible organic electronics. *Nature communications* **9**, 1-9 (2018).

43. Kitahara, G. *et al.* Meniscus-controlled printing of single-crystal interfaces showing extremely sharp switching transistor operation. *Science advances* **6**, eabc8847 (2020).

44. Waldrip, M., Jurchescu, O. D., Gundlach, D. J. & Bittle, E. G. Contact resistance in organic field-effect transistors: conquering the barrier. *Advanced Functional Materials* **30**, 1904576 (2020).

45. Wang, C. *et al.* Thiol–ene cross-linked polymer gate dielectrics for low-voltage organic thin-film transistors. *Chemistry of Materials* **25**, 4806-4812 (2013).

46. Blülle, B., Häusermann, R. & Batlogg, B. Approaching the trap-free limit in organic single-crystal field-effect transistors. *Physical Review Applied* **1**, 034006 (2014).

47. Kim, S. H., Jang, M., Yang, H., Anthony, J. E. & Park, C. E. Physicochemically Stable Polymer-Coupled Oxide Dielectrics for Multipurpose Organic Electronic Applications. *Advanced Functional Materials* **21**, 2198-2207 (2011).

48. Zhao, J., Tang, W., Li, Q., Liu, W. & Guo, X. Fully solution processed bottom-gate organic field-effect transistor with steep subthreshold swing approaching the theoretical limit. *IEEE Electron Device Letters* **38**, 1465-1468 (2017).

49. Ortiz, R. P., Facchetti, A. & Marks, T. J. High-k organic, inorganic, and hybrid dielectrics for low-voltage organic field-effect transistors. *Chemical reviews* **110**, 205-239 (2010).

50. Zhou, Z. *et al.* Field-effect transistors based on 2D organic semiconductors developed by a hybrid deposition method. *Advanced Science* **6**, 1900775 (2019).
51. Peng, B., Huang, S., Zhou, Z. & Chan, P. K. L. Solution-Processed Monolayer Organic Crystals for High-Performance Field-Effect Transistors and Ultrasensitive Gas Sensors. *Advanced Functional Materials* **27**, 1700999 (2017).
52. Kang, M. J. *et al.* Alkylated Dinaphtho [2, 3-b: 2′, 3′-f] Thieno [3, 2-b] Thiophenes (Cn-DNTTs): organic semiconductors for high-performance thin-film transistors. *Advanced Materials* **23**, 1222-1225 (2011).
53. Minemawari, H., Yamada, T. & Matsui, H. J. y. Tsutsumi, S. Haas, R. Chiba, R. Kumai and T. Hasegawa. *Nature* **475**, 364-367 (2011).
54. Chen, M., Peng, B., Huang, S. & Chan, P. K. L. Understanding the Meniscus-Guided Coating Parameters in Organic Field-Effect-Transistor Fabrications. *Advanced Functional Materials* **30**, 1905963 (2020).
55. He, D. *et al.* Ultrahigh mobility and efficient charge injection in monolayer organic thin-film transistors on boron nitride. *Science advances* **3**, e1701186 (2017).
56. Uemura, T., Hirose, Y., Uno, M., Takimiya, K. & Takeya, J. Very high mobility in solution-processed organic thin-film transistors of highly ordered [1] benzothieno [3, 2-b] benzothiophene derivatives. *Applied Physics Express* **2**, 111501 (2009).
57. Cho, J.-m. & Mori, T. Low-temperature band transport and impact of contact resistance in organic field-effect transistors based on single-crystal films of Ph-BTBT-C10. *Physical Review Applied* **5**, 064017 (2016).

58. Wu, H., Iino, H. & Hanna, J.-i. Scalable Ultrahigh-Speed Fabrication of Uniform Polycrystalline Thin Films for Organic Transistors. *ACS Applied Materials & Interfaces* **12**, 29497-29504 (2020).

59. Chen, Z. *et al.* Organic Semiconductor Crystal Engineering for High-Resolution Layer-Controlled 2D Crystal Arrays. *Advanced Materials*, 2104166 (2021).

60. Liu, C. *et al.* Controlling the crystal formation in solution-process for organic field-effect transistors with high-performance. *Organic Electronics* **13**, 2975-2984 (2012).

61. Tsutsui, Y. *et al.* Unraveling Unprecedented Charge Carrier Mobility through Structure Property Relationship of Four Isomers of Didodecyl [1] benzothieno [3, 2-b][1] benzothiophene. *Advanced materials* **28**, 7106-7114 (2016).

62. Yuan, Y. *et al.* Ultra-high mobility transparent organic thin film transistors grown by an off-centre spin-coating method. *Nature communications* **5**, 1-9 (2014).

63. Minari, T. *et al.* Room-Temperature Printing of Organic Thin-Film Transistors with π-Junction Gold Nanoparticles. *Advanced Functional Materials* **24**, 4886-4892 (2014).

64. Watanabe, S. *et al.* Remarkably low flicker noise in solution-processed organic single crystal transistors. *Communications Physics* **1**, 1-8 (2018).

65. Yokota, T. *et al.* A few-layer molecular film on polymer substrates to enhance the performance of organic devices. *Nature Nanotechnology* **13**, 139-144 (2018).

66. Zhang, Z. *et al.* Direct Patterning of Self-Assembled Monolayers by Stamp Printing Method and Applications in High Performance Organic Field-Effect Transistors and Complementary Inverters. *Advanced Functional Materials* **25**, 6112-6121 (2015).

67. Sugiyama, M. *et al.* An ultraflexible organic differential amplifier for recording electrocardiograms. *Nature Electronics* **2**, 351-360, doi:10.1038/s41928-019-0283-5 (2019).

68. Jinno, H. *et al.* Low operating voltage organic transistors and circuits with anodic titanium oxide and phosphonic acid self-assembled monolayer dielectrics. *Organic Electronics* **40**, 58-64 (2017).

69. Park, J. H., Jung, E. H., Jung, J. W. & Jo, W. H. A fluorinated phenylene unit as a building block for high-performance n-type semiconducting polymer. *Advanced Materials* **25**, 2583-2588 (2013).

70. Sekitani, T., Zschieschang, U., Klauk, H. & Someya, T. Flexible organic transistors and circuits with extreme bending stability. *Nature materials* **9**, 1015-1022 (2010).

71. Sun, B., Hong, W., Yan, Z., Aziz, H. & Li, Y. Record high electron mobility of 6.3 cm2V− 1s− 1 achieved for polymer semiconductors using a new building block. *Advanced Materials* **26**, 2636-2642 (2014).

72. Kumagai, S. *et al.* Coherent Electron Transport in Air-Stable, Printed Single-Crystal Organic Semiconductor and Application to Megahertz Transistors. *Advanced Materials* **32**, 2003245 (2020).

73. Oh, J. H., Wei, P. & Bao, Z. Molecular n-type doping for air-stable electron transport in vacuum-processed n-channel organic transistors. *Applied Physics Letters* **97**, 269 (2010).

74. Matthews, J. R. *et al.* Scalable synthesis of fused thiophene-diketopyrrolopyrrole

semiconducting polymers processed from nonchlorinated solvents into high performance thin film transistors. *Chemistry of Materials* **25**, 782-789 (2013).

75. Lee, W. Y. *et al.* Effect of Non-Chlorinated Mixed Solvents on Charge Transport and Morphology of Solution-Processed Polymer Field-Effect Transistors. *Advanced Functional Materials* **24**, 3524-3534 (2014).
76. Giri, G., Park, S., Vosgueritchian, M., Shulaker, M. M. & Bao, Z. High-mobility, aligned crystalline domains of TIPS-pentacene with metastable polymorphs through lateral confinement of crystal growth. *Advanced materials* **26**, 487-493 (2014).
77. Uno, M., Cha, B.-S., Kanaoka, Y. & Takeya, J. High-speed organic transistors with three-dimensional organic channels and organic rectifiers based on them operating above 20 MHz. *Organic Electronics* **20**, 119-124 (2015).
78. Steudel, S. *et al.* 50 MHz rectifier based on an organic diode. *Nature materials* **4**, 597-600 (2005).
79. Steudel, S. *et al.* Comparison of organic diode structures regarding high-frequency rectification behavior in radio-frequency identification tags. *Journal of applied physics* **99**, 114519 (2006).
80. Ai, Y. *et al.* 14 MHz organic diodes fabricated using photolithographic processes. *Applied physics letters* **90**, 262105 (2007).
81. Wang, H. *et al.* Interface effect on the performance of rectifier based on organic diode. *IEEE Electron Device Letters* **31**, 506-508 (2010).
82. Lin, C.-Y. *et al.* High-frequency polymer diode rectifiers for flexible wireless power-

transmission sheets. *Organic Electronics* **12**, 1777-1782 (2011).

83. Heljo, P., Lilja, K. E., Majumdar, H. S. & Lupo, D. High rectifier output voltages with printed organic charge pump circuit. *Organic Electronics* **15**, 306-310 (2014).

84. Viola, F. A. *et al.* A 13.56 MHz rectifier based on fully inkjet printed organic diodes. *Advanced Materials* **32**, 2002329 (2020).

85. Li, T. *et al.* Integrated molecular diode as 10 MHz half-wave rectifier based on an organic nanostructure heterojunction. *Nature communications* **11**, 1-10 (2020).

86. Ferchichi, K., Pecqueur, S., Guerin, D., Bourguiga, R. & Lmimouni, K. High Rectification Ratio in Polymer Diode Rectifier through Interface Engineering with Self-Assembled Monolayer. *Electronic Materials* **2**, 445-453 (2021).

87. Steudel, S., Myny, K., Vicca, P., Cheyns, D. & Heremans, P. in *2008 IEEE International Electron Devices Meeting.*

88. Kang, C. m. *et al.* 1 GHz Pentacene Diode Rectifiers Enabled by Controlled Film Deposition on SAM-Treated Au Anodes. *Advanced Electronic Materials* **2**, 1500282 (2016).

89. Im, D., Moon, H., Shin, M., Kim, J. & Yoo, S. Towards Gigahertz Operation: Ultrafast Low Turn-on Organic Diodes and Rectifiers Based on C60 and Tungsten Oxide. *Advanced Materials* **23**, 644-648 (2011).
